# Stem loop binding protein promotes SARS-CoV-2 replication via -1 programmed ribosomal frameshifting

**DOI:** 10.1038/s41392-025-02277-w

**Published:** 2025-06-13

**Authors:** Tanxiu Chen, Ruimin Zhu, Tingfu Du, Hao Yang, Xintian Zhang, Zhixing Wang, Yong Zhang, Wenqi Quan, Bin Yin, Yunpeng Liu, Shuaiyao Lu, Xiaozhong Peng

**Affiliations:** 1 State Key Laboratory of Respiratory Health and Multimorbidity, National Center of Technology Innovation for Animal Model, Key Laboratory of Pathogen Infection Prevention and Control (Peking Union Medical College), Ministry of Education, Institute of Laboratory Animal Science, CAMS & PUMC, Beijing, China; 2 Institute of Medical Biology, Chinese Academy of Medical Sciences and Peking Union Medical College, Kunming, China; 3 Institute of Neurology and Department of Neurology, Jiangxi Academy of Clinical Medical Sciences, Jiangxi Key Laboratory of Neurological Diseases, The First Affiliated Hospital, Jiangxi Medical College, Nanchang University, Nanchang, China; 4 Department of Molecular Biology and Biochemistry, Institute of Basic Medical Sciences, Medical Primate Research Center, Neuroscience Center, CAMS & PUMC, Beijing, China; 5 Key Laboratory of Pathogen Infection Prevention and Control (Peking Union Medical College), Ministry of Education, Beijing, China; 6 State Key Laboratory of Respiratory Health and Multimorbidity, Beijing, China

**Keywords:** Target identification, Gene therapy

## Abstract

The -1 programmed ribosomal frameshifting (-1 PRF) in severe acute respiratory syndrome coronavirus 2 (SARS-CoV-2) is crucial for keeping the balance between pp1a and pp1ab polyproteins. To date, the host factors influencing this process remain poorly understood. Using RNA pull-down assays combined with mass spectrometry screening, we discovered five host proteins interacting with -1 PRF RNA, including Stem Loop Binding Protein (SLBP). Our findings revealed that SLBP overexpression enhanced frameshifting and promoted viral replication. Moreover, the interaction between SLBP and -1 PRF RNA was predicted using the PrismNet deep learning tool, which calculated a high binding probability of 0.922. Using Electrophoretic Mobility Shift Assays (EMSAs) and RNA pull down assays, our findings demonstrated SLBP’s direct binding to the SARS-CoV-2 genome, with preferential affinity for the stem loop 3 region of the -1 PRF RNA. Using smFISH assays, we further confirmed their physical colocalization. The role of SLBP in promoting frameshifting was verified using an in vitro translation system. Further investigation showed that SLBP deletions reshaped the host factor pattern around -1 PRF RNA, diminishing interactions with FUBP3 and RPS3A while enhancing RPL10A binding. Together, our findings identify SLBP as a host protein that promotes SARS-CoV-2 frameshifting, highlighting its potential as a druggable target for COVID-19.

## Introduction

As of April 2024, according to data provided by Worldometers (www.worldometers.info/coronavirus/), COVID-19 cases have exceeded 704 million with 7.01 million fatalities. The mortality rate of the disease, calculated based on reported cases, remains distressing. Currently, research on COVID-19 treatments is centered on compounds that interact with non-structural proteins (NSPs), including NSP3 (PLpro), NSP5 (3CLpro), and NSP12 (RdRp).^1^ The efficacy of approved drugs in managing severe illness remains limited. Paxlovid, a first-line treatment for COVID-19, demonstrates therapeutic benefit exclusively in mild-to-moderate cases, and its use may lead to relapse.^2^ A more comprehensive elucidation of the molecular mechanisms underpinning Severe Acute Respiratory Syndrome Coronavirus 2 (SARS-CoV-2) infection is paramount for the successful development and implementation of targeted therapeutic strategies to combat COVID-19.

In eukaryotic organisms, most proteins are encoded by a single reading frame within the genetic material, and only a few eukaryotic mRNAs have been reported to encode proteins in overlapping reading frames,^3^ including embryonal carcinoma differentiation regulated gene (Edr),^4^ paraneoplastic antigen (Ma3),^5^ and human immunodeficiency virus (HIV) cytokine receptor (ccr5),^6^ and interleukin receptor subunits (ILR).^6^ Overlapping reading frames enable multiple protein production in certain pathogenic viruses. In 1985, Jacks and Varmus discovered that p108 Gag-Pol protein could be generated in an in vitro translation system by cloning the full-length sequence of *Gag-Pol* from the Rous Sarcoma Virus onto a plasmid with the SP6 promoter, confirming the existence of programmed ribosomal frameshifting (PRF) in viral protein translation.^7^ To date, -1 PRF is the most well-known form, as demonstrated in HIV and infectious bronchitis virus—the first demonstrated coronavirus.^8^ These viruses trigger a ribosomal frameshift, causing ribosomes to shift back by one nucleotide near the 5’ end of the open reading frame. This -1 slippage occurs through a combination of a specialized slippery nucleotide sequence and an adjacent stem-loop structure, allowing translation to proceed in the alternative reading frame. Coronavirus such as SARS-CoV-2 have a conserved -1 PRF site, which plays a key role in regulating programmed ribosomal frameshift. This mechanism precisely regulates the production of NSPs, which are indispensable for the virus’s ability to replicate.^9^ Through a dual fluorescence reporter screening approach, researchers have pinpointed merafloxacin, a fluoroquinolone antibiotic, as a potent suppressor of -1 programmed ribosomal frameshifting (-1 PRF) in beta coronaviruses, including SARS-CoV-2.^10^ The carbamycin disrupts viral replication by specifically targeting the pseudoknot structure in the -1 PRF RNA, thereby impeding the frameshift mechanism critical for producing viral transcription and replication machinery.^11^ Consequently, SARS-CoV-2 -1 PRF RNA has emerged as a promising therapeutic target.

In SARS-CoV-2, ORF1a and ORF1b partially overlap. As ORF1b lacks its own translation start codon, polyprotein pp1ab (NSP1-16), a *trans*-frame fusion, is produced via -1 PRF, forming NSP12-16 after self-encoded enzyme cleavage in excess compared with the polyprotein pp1a (NSP1-11).^12^ During viral infection, The ORF1b-encoded replicative enzymes (NSP12-16) of SARS-CoV play significant roles in promoting viral RNA replication.^13^ Additionally, -1 PRF in SARS-related coronaviruses occurs with relatively high efficiency (25% to 75%).^14^ When frameshift efficiency changes, the ratio of pp1a and pp1ab proteins becomes unbalanced, potentially affecting the SARS-CoV-2 life cycle and ultimately resulting in abnormal production of genomic and subgenomic RNA and virus particle assembly.^15^ SARS-CoV-2 contains three RNA structural elements for -1 PRF: an attenuator loop, a slippery sequence (UUUAAAC), and a stimulatory pseudoknot containing three stem loops (SLs).^14^ The -1 Frameshifting Element (-1 FSE), comprising a slippery sequence and a pseudoknot, plays a key role in balancing pp1a and pp1ab expression levels.^16^ It has long been known that RNA never exists in a completely naked state.^17^ Similarly, -1 frameshifting events always involve interactions among host factors, viral proteins, and specific sequences. In porcine reproductive and respiratory syndrome virus (PRRSV), NSP1β and PCBP facilitate ribosomal frameshifting via interactions with defined regions proximal to the slippery sequence. In encephalomyocarditis virus infection, the viral 2 A protein assumes a pivotal role in mediating ribosomal frameshifting.^18^ In SARS-CoV-2, research has demonstrated that the short isoform of the zinc-finger antiviral protein (ZAP-S) functions as an antiviral factor that inhibits -1 PRF by physical interaction with the FSE.^19^ Eukaryotic translation initiation factor 2 A (eIF2A) promotes SARS-CoV-2 replication within host cells by directly and specifically increasing -1 PRF.^20^ Despite biochemical provides understanding the critical role of -1 PRF for SARS-CoV-2, the underlying mechanisms and involved host cellular factors regulating viral frameshifting efficiency remain poorly characterized. Therefore, to better understand how SARS-CoV-2 induces frameshifting, the -1 PRF RNA‒protein interaction network requires systematic study.

In this study, our work focused on a -1 PRF RNA direct binding screening approach to identify modulators influencing frameshifting in SARS-CoV-2. Through this approach, we identified five proteins—Far Upstream Element Binding Protein 3 (FUBP3), Stem Loop Binding Protein (SLBP), Ribosomal Protein L10A (RPL10A), Ribosomal Protein S3A (RPS3A), and Ribosomal Protein S14 (RPS14)—as interaction partners of -1 PRF RNA that promote SARS-CoV-2 replication by regulating frameshifting. A complex web of interactions formed among these host factors. Our results show that SLBP knockdown selectively modified host protein interactions with -1 PRF RNA, characterized by diminished FUBP3 and RPS3A binding but enhanced RPL10A engagement. Moreover, we revealed the binding motifs of -1 PRF RNA with these host factors. We demonstrated that SLBP acts as a critical scaffold protein among the five host factors and regulates the -1 PRF process. Demonstration of SLBP/-1 PRF RNA interaction led to our recognition of SLBP as a potential antiviral target.

## Results

### Five host factors interacting with SARS-CoV-2 -1 PRF RNA were identified

**Fig. 1:**
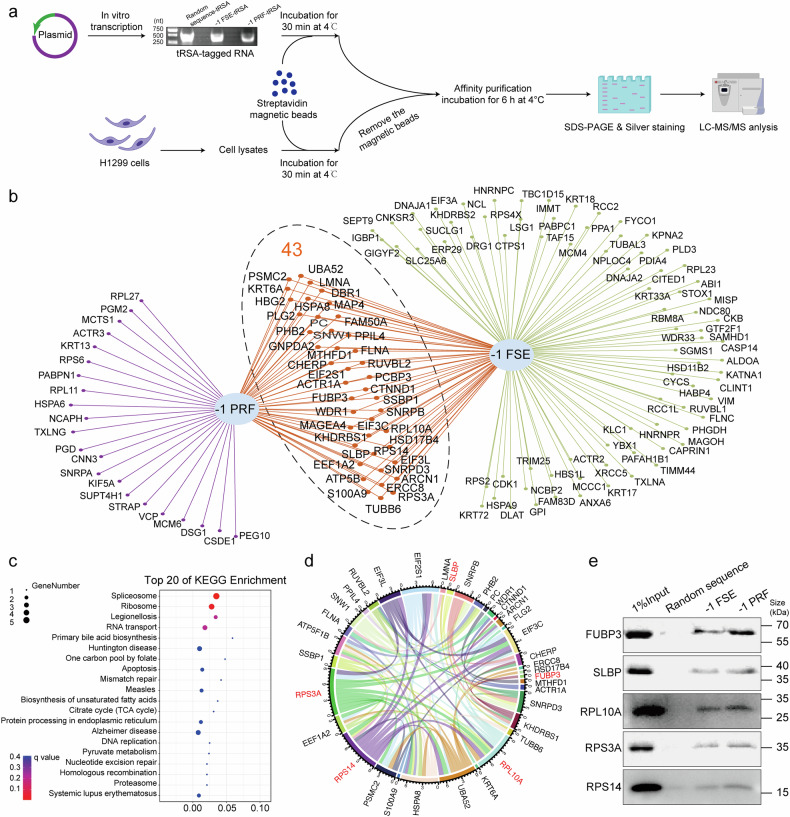
Identification of host factors binding with SARS-CoV-2 -1 PRF RNA. **a** Schematic overview of the method for capturing protein interactors of SARS-CoV-2 -1 FSE RNA and -1 PRF RNA. RNA probes were produced by in vitro transcription. In vitro transcription-synthesized RNA probes were captured onto streptavidin-coated magnetic beads. Following capture on streptavidin beads, RNA probes were immediately incubated with total cell lysates of H1299 cells removed nonspecific binding by incubating with streptavidin beads. Proteins were captured by RNA probes, pulled down and subjected to mass spectrometry. **b** Overlap in the Venn diagram showing host factors interacting with -1 FSE RNA and -1 PRF RNA. **c** KEGG pathway enrichment analysis. **d** The top 30 hub genes of the candidate host factors. **e** The RNA pull-down assay confirmed the ability of FUBP3, SLBP, RPL10A, RPS3A, and RPS14 to interact with -1 FSE RNA and -1 PRF RNA in H1299 total cell lysates. Experiments were repeated three times with similar results

To profile of cellular proteins associated with SARS-CoV-2 -1 PRF RNA, we employed RNA pull-down assays followed by streptavidin-conjugated magnetic beads capture. The tRNA scaffold to a streptavidin aptamer (tRSA) sequence has been reported to efficiently pull down RNA-binding proteins from cell lysates.^21^ To rigorously control for non-specific interactions and enhance the fidelity of our dataset, both -1 PRF and -1 FSE RNA probes tagged with the tRSA sequence were utilized in parallel screens to capture binding proteins. As a random sequence control for nonspecificity, cellular protein lysates derived from H1299 cells were incubated with tRSA- conjugated -1 PRF and -1 FSE RNA probes. Streptavidin magnetic beads were used to pull down proteins bound to the tRSA-labeled probes. Protein samples were resolved using SDS‒PAGE followed by silver staining detection. Bands with detectable protein content were cut from gels, trypsin-digested, then analyzed via liquid chromatography tandem mass spectrometry (LC‒MS/MS) (Fig. 1a). We identified 122 proteins (Data set 1) that interacted with -1 FSE RNA and 65 proteins (Data set 1) that interacted with -1 PRF RNA compared to the random sequence. Using overlap analysis, we confirmed the screening results and narrowed our focus to 43 high-confidence candidates (Data set 1) that showed specific interactions with -1 PRF and -1 FSE RNA baits (Fig. 1b). KEGG pathway enrichment analysis demonstrated enrichment for 43 RNA binding proteins involved in several pathways, including the process of spliceosome and ribosome function (Fig. 1c). Protein‒protein interaction network was generated through STRING database analysis. We then employed CytoHubba, a Cytoscape plugin, to identify hub genes. Based on node degree, we identified hub genes among these candidate host factors. For better visualization, the interactions of the top 30 hub genes were reconstructed using Cytoscape software (Fig. 1d). We performed a literature search to examine whether these host factors were biologically related to viral replication. Twelve genes were selected for further analysis: RPS3A, RPS14, RPL10A, HSPA8, EIF2S1, EEF1A2, UBA52, EIF3C, PHB2, EIF3L, FUBP3, and SLBP. RNA pull-down Western blot analysis showed that five proteins—FUBP3, SLBP, RPL10A, RPS3A, and RPS14—interacted with -1 FSE and -1 PRF RNA, consistent with the LC‒MS/MS results (Fig. 1e). In contrast, HSPA8, EIF2S1, EEF1A2, UBA52, EIF3C, PHB2, and EIF3L showed low binding capacity to -1 PRF RNA. Among the five interacting host factors, RPL10A is recognized as a component of the 60S ribosomal subunit, while RPS3A and RPS14 are both components of the 40S ribosomal subunit,^22^ further implicating the ribosome in the viral life cycle. FUBP3 has been reported to bind to the internal ribosome entry site of enterovirus 71,^23^ regulate innate immunity and influence virus replication.^24^ SLBP is an RNA stem loop binding protein involved in viral replication.^25^ These findings suggest that the identified proteins play critical roles in the host response to SARS-CoV-2 infection and could serve as potential therapeutic targets for the development of antiviral treatments.

### Functional screen of host factors interacting with SARS-CoV-2 -1 PRF RNA

**Fig. 2:**
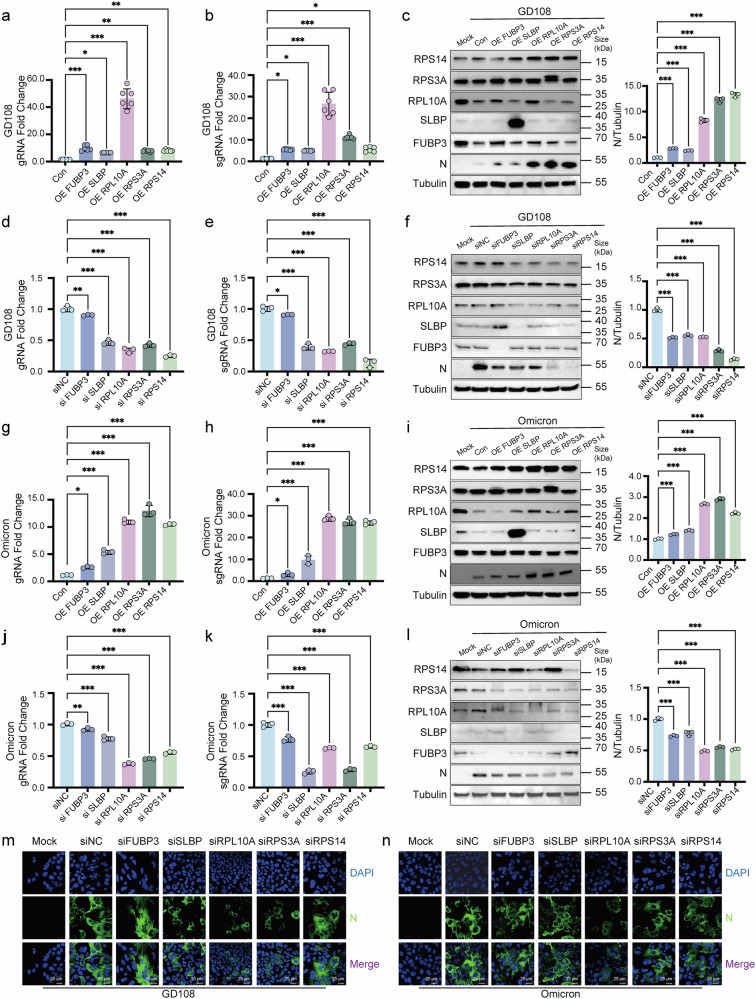
Abnormal expression of FUBP3, SLBP, RPL10A, RPS3A, and RPS14 affected SARS-CoV-2 replication. Overexpression of host factors increased the expression levels of gRNA and sgRNA of SARS-CoV-2 in Huh7 cells infected with the GD108 strain (MOI = 0.05, 48 h) (**a**, **b**, *n* = 6, **c**, *n* = 3). Knockdown of host factors decreased the expression levels of gRNA and sgRNA of SARS-CoV-2 in Huh7 cells infected with the GD108 strain (MOI = 0.05, 48 h) (**d**–**f**, *n* = 3). Overexpression of host factors increased the expression levels of gRNA and sgRNA of SARS-CoV-2 in Huh7 cells infected with the Omicron strain (MOI = 0.05, 48 h) (**g**–**i**, *n* = 3). Knockdown of host factors decreased the expression levels of gRNA and sgRNA of SARS-CoV-2 in Huh7 cells infected with the Omicron strain (MOI = 0.05, 48 h) (**j**–**l**, *n* = 3). **m**, **n** Knockdown of host factors decreased the N protein expression level in Huh7 cells infected with GD108 and Omicron strains using laser confocal microscopy. Scale bars, 25 μm. For all figures, experiments were repeated at least three times with similar results. Data are represented as the mean ± SEM. Multiple comparisons were performed using ANOVA with Dunnett’s test. *P < 0.05, **P < 0.01, and ***P < 0.001

Through our investigation, we identified FUBP3, SLBP, RPL10A, RPS3A, and RPS14 as host factors that interact with -1 PRF RNA. Based on their interactions, we hypothesized that these host factors could play either synergistic or antagonistic roles in modulating SARS-CoV-2 replication. To test this hypothesis, we systematically manipulated the expression levels of these factors and evaluated their impact on viral replication. We found that overexpression of FUBP3, SLBP, RPL10A, RPS3A, and RPS14 accelerated SARS-CoV-2 propagation and increased nucleocapsid protein abundance (Fig. 2a–c, g–i). Conversely, Silencing these genes reduced viral propagation in supernatant samples collected at 48 h post infection (hpi), including both genome RNA (gRNA) and subgenome RNA (sgRNA), resulting in reduced nucleocapsid protein levels (Fig. 2d–f, j–l). To ensure the robustness of our observations, we extended our experiments to H1299 cell lines, where we obtained consistent results (Supplementary Fig. S1a–d). Additionally, we used immunofluorescence assays to examine viral nucleoprotein expression using anti-N antibody at 48 hpi. Our results confirmed that FUBP3, SLBP, RPL10A, RPS3A, and RPS14 knockdown in Huh7 cells exposed to SARS-CoV-2 reduced nucleoprotein expression compared to the negative control group (Fig. 2m, n). Comparable results emerged upon infection with varied SARS-CoV-2 isolates, encompassing GD108 and Omicron. Collectively, our data demonstrate that FUBP3, SLBP, RPL10A, RPS3A, and RPS14 function as pro-viral host factors that facilitate SARS-CoV-2 proliferation in the tested cellular model. These findings provide new insights into host-pathogen interactions and highlight potential targets for antiviral therapeutic development.

### Abnormal expression of host factors affects SARS-CoV-2 frameshifting

**Fig. 3:**
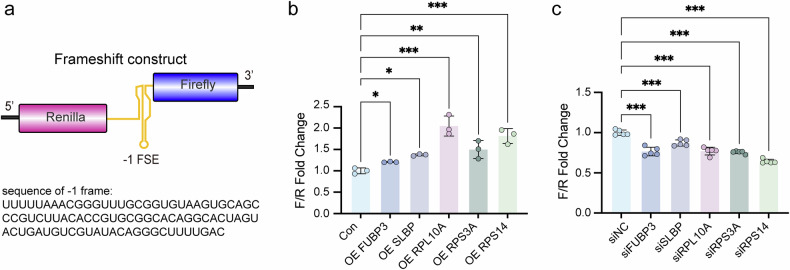
The expression of five host factors promoted SARS-CoV-2 frameshifting. **a** Schematic representation of the luciferase frameshift reporter construct. Renilla luciferase and Firefly luciferase were separated by the SARS-CoV-2 -1 FSE sequence. The ratio of Firefly to Renilla luciferase (F/R) was used to quantify the level of frameshifting. **b** The F/R ratio of HEK293T cells transfected with the pHRF-FSE(-1) luciferase reporter vector were cotransfected with the empty vector (Con) or gene overexpression vector (*n* = 3). **c** The F/R ratio of HEK293T cells transfected with the pHRF-FSE(-1) luciferase reporter vector were cotransfected with the negative control siRNA or gene knockdown siRNA (*n* = 5). For all figures, experiments were repeated at least three times with similar results. Data points represent the mean ± SEM. Multiple comparisons were performed using ANOVA with Dunnett’s test. *P < 0.05, **P < 0.01, and ***P < 0.001

To determine whether the identified host factors (FUBP3, SLBP, RPL10A, RPS3A, and RPS14) influence -1 programmed ribosomal frameshifting (-1 PRF), we designed a dual luciferase reporter system, pHRF-FSE(-1), which enables quantitative measurement of frameshifting efficiency (Fig. 3a). In this construct, the upstream ORF encoding Renilla luciferase contains a normal ATG initiation codon in the 0 frame, while the downstream ORF encoding Firefly luciferase lacks an initiation codon and depends on SARS-CoV-2 -1 PRF occurring in the -1 frame for expression. When we overexpressed FUBP3, SLBP, RPL10A, RPS3A, or RPS14 in transfected cells, we observed a significant increase in the Firefly/Renilla luciferase ratio (Fig. 3b), indicating that these host factors promote -1 PRF efficiency. Conversely, silencing of FUBP3, SLBP, RPL10A, RPS3A, or RPS14 in 293 T cells led to a reduction in the Firefly/Renilla ratio (Fig. 3c), confirming that these factors are functionally involved in modulating frameshifting efficiency. Our results demonstrated that these host factors promote SARS-CoV-2 frameshifting. Together, these findings support our hypothesis that FUBP3, SLBP, RPL10A, RPS3A, and RPS14 are potential frameshifting regulatory factors.

### Identifying SLBP as an important frameshifting regulating factor of SARS-CoV-2

**Fig. 4:**
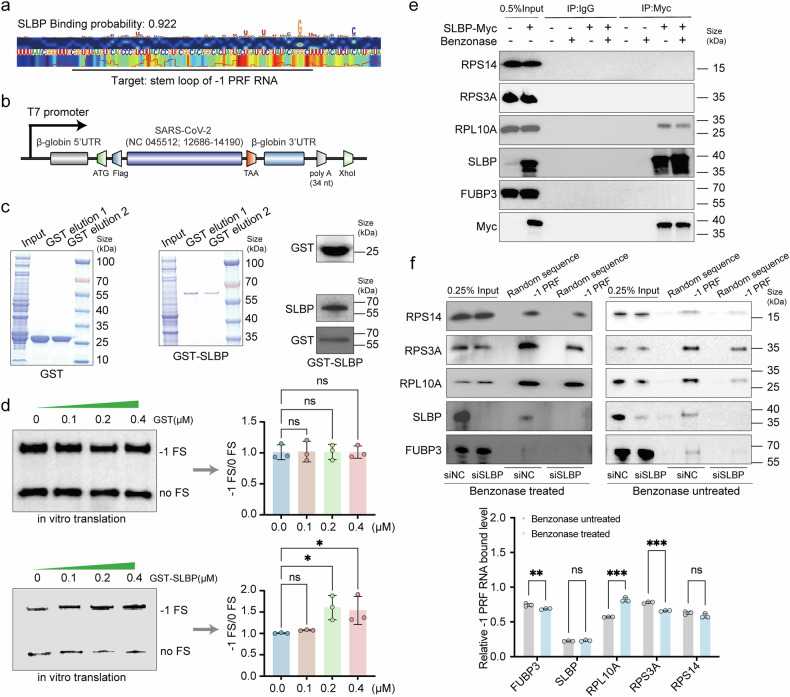
SLBP is a core host factor involved in SARS-CoV-2 frameshifting. **a** Applying the PrismNet deep learning tool accurately predicts SLBP that bind to SARS-CoV-2. Saliency maps from PrismNet showing the predicted binding site of SLBP with predicted binding probabilities shown at the top. The black line indicates the region of the stem loop of -1 PRF RNA. Green rectangles show predicted strong binding sites, and orange rectangles show relatively weaker binding sites. **b** The vector contained the T7 promoter, β-globin 5′ UTR and 3′ UTR as well as a 34 nt long poly-(A) tail. The insert coding sequence was derived from nucleotides 12686-14190 of SARS-CoV-2 (NC 045512), and the Flag-tag was introduced at the N-terminus to facilitate detection. The schematic diagram was generated with IBS 2.0 (https://ibs.renlab.org/#/server). **c** The purified protein was characterized using SDS‒PAGE and Western blotting. **d** The in vitro translation assay was performed. Flag-tagged peptides generated by ribosomes that do not have frameshifts (no FS) or that enter the -1 reading frame (-1 FS) were identified via western blotting using an anti-Flag antibody (*n* = 3). **e** The coimmunoprecipitation (co-IP) assay confirmed the interaction among SLBP, FUBP3, RPL10A, RPS3A, and RPS14. Benzonase (150 U/ml) was added where indicated. **f** We tested whether SLBP affected the binding of the other four host factors to -1 PRF RNA by conducting RNA pull-down WB assays after SLBP knockdown in H1299 cells. Benzonase (150 U/ml) was added where indicated (*n* = 3). Experiments were repeated three times with similar results. Data points represent the mean ± SEM. Multiple comparisons were performed using ANOVA with Dunnett’s test. *P < 0.05, **P < 0.01, and ***P < 0.001

Among all five host factors, RPL10A, RPS3A, and RPS14 are ribosomal-related proteins, while FUBP3 and SLBP are non-ribosomal-related proteins. There are few reports on the impact of non-ribosomal-related proteins on frameshift. Thus, we focused on FUBP3 and SLBP. It has been shown that the 3’UTR of JEV is commonly recognized by FUBP3 to regulate its replication.^26^ FUBP3 can bind to the 5’UTR of EV71, thereby enhancing IRES activity.^23^ SLBP regulates histone mRNA posttranscriptionally, influencing pre-mRNA processing, nuclear export, stability, and translation. SLBP contains an RNA binding domain (RBD), and it was previously reported that SLBP expression inhibits HIV replication^27^ while promoting HCMV replication.^25^ NSP14 was reported to disrupt the recruitment of SLBP to pre-mRNAs.^28^ Additionally, PrismNet was applied to predict SLBP-SARS-CoV-2 -1 PRF RNA binding affinity. Consistent with the western blotting results, the binding probability between SLBP and stem loops (SLs) of -1 PRF RNA reached a high affinity of approximately 92.2% (Fig. 4a). Unfortunately, since the other four host factors are not in the database, PrismNet predictions were not made for them. Furthermore, our findings indicated that SLBP expression exerted no substantial influence on cell proliferation in both Huh7 and H1299 cells (Supplementary Fig. S2a–d). Due to its high binding probability, we chose to concentrate on SLBP for further studies. Given its strong affinity, we decided to focus on SLBP for more in-depth research. To delve into the specificity of SLBP in SARS-CoV-2 replication, we also examined whether the presence of SLBP impacts the replication of other coronaviruses. Our results showed SLBP silencing also inhibited HCoV-229E replication (Supplementary Fig. S3a, b). Following SLBP knockdown, viral plaque formation was approximately 0.35-fold that of the control group, and the expression of 229E N protein was reduced by approximately 2-fold. Additionally, the SLBP protein also interacted with 229E -1 PRF RNA (Supplementary Fig. S3c). We further analyzed the effect of SLBP on frameshifting utilizing the rabbit reticulocyte lysate (RRL) translation system in vitro. First, a Flag-labeled pcDNA4.0 vector incorporating SARS-CoV-2 genomic region 12686-14190 was created as a frameshift model (Fig. 4b). Then, as illustrated in Fig. 4c, recombinant GST-SLBP was expressed and purified. In contrast to GST, which had no detectable impact on frameshifting, the recombinant GST-SLBP enhanced frameshifting efficiency. During the titration of escalating quantities of GST-SLBP, we noted a concomitant rise in the frameshift product (Fig. 4d). In accordance with luciferase assay results, this indicated that SLBP directly targeted the native SARS-CoV-2 -1 PRF RNA.

Because all five host factors interacted with -1 PRF RNA, we investigated whether there are interactions among FUBP3, SLBP, RPL10A, RPS3A, and RPS14. To address this question, we performed co-immunoprecipitation studies to examine their physical interactions. We conducted immunoprecipitation of Myc followed by immunoblotting against FUBP3, SLBP, RPL10A, RPS3A, and RPS14. RPL10A, RPS3A, and RPS14 specifically co-immunoprecipitated with Myc-tagged FUBP3 (Supplementary Fig. S4a). FUBP3, SLBP, RPS3A, and RPS14 specifically coimmunoprecipitated with Myc-tagged RPL10A (Supplementary Fig. S4b). FUBP3, RPL10A, and RPS14 specifically coimmunoprecipitated with Myc-tagged RPS3A (Supplementary Fig. S4c). FUBP3, RPL10A, and RPS3A specifically coimmunoprecipitated with Myc-tagged RPS14 (Supplementary Fig. S4d). In order to eliminate protein interactions that are indirectly mediated by DNA bridging, benzonase nuclease was added to the extract in order to remove any leftover DNA. Following benzonase treatment, nucleic acids of whole-cell lysates were confirmed to be removed (Supplementary Fig. S5). We found that RPL10A specifically coimmunoprecipitated with Myc-tagged SLBP (Fig. 4e).

To analyze the molecular weights of -1 PRF RNA pull-down complexes, we resolved the pull-down products by Native-PAGE and performed immunodetection using antibodies against SLBP, FUBP3, RPL10A, RPS3A, and RPS14. Immunodetection with antibodies specific for each factors confirmed comparable complex abundance and resolution in -1 PRF RNA pull-down complexes. SLBP-containing complexes were identified at around 100 kDa, 230 kDa, and 650 kDa. FUBP3-containing complexes were detected at above 100 kDa. RPL10A-containing complexes appeared near 100 kDa, and 650 kDa. RPS3A-containing complexes were detected at approximately 100 kDa, 230 kDa, and above 670 kDa. RPS14-containing complexes were detected at approximately 230 kDa (Supplementary Fig. S6). Based on the experimental results described above, six distinct protein complexes with varying molecular weights were identified: the SLBP-RPL10A complex (~93 kDa), the RPS3A-? complex (>100 kDa), the FUBP3-?? complex (>100 kDa), the SLBP-RPS3A-RPS14-??? complex (~230 kDa), the SLBP-RPL10A-???? complex (~650 kDa), and the RPS3A-????? complex (>670 kDa) (Supplementary Fig. S6). These results indicate that the these five host factors participate in -1 PRF regulation by forming complexes with -1 PRF RNA. Notably, our study did not detect an independent FUBP3-SLBP-RPL10A-RPS3A-RPS14 complex (predicted to be ~164 kDa), we hypothesize that these complexes may undergo conditional dissociation or reorganization, potentially resulting in a molecular weight below or exceeding the expected 164 kDa.

We further examined whether SLBP expression influences the relationships among the other four host factors and SARS-CoV-2 -1 PRF RNA. To test this possibility, we conducted an RNA pull-down assay using whole-cell lysates from SLBP-silenced H1299 cells. Silencing of SLBP in H1299 cells significantly reduced the interaction of particular host factors with SARS-CoV-2 -1 PRF RNA in comparison to the control group (Fig. 4f). Benzonase treatment outcomes show cellular nucleic acids’ involvement in modulating the association of particular host proteins with -1 PRF RNA. The reduced binding of FUBP3 and RPS3A, coupled with enhanced RPL10A association, suggests nucleic acid-mediated modulation of these interactions. However, the unchanged binding of SLBP and RPS14 indicates their endogenous nucleic acid-independent engagement with -1 PRF RNA. Collectively, SLBP expression levels regulate the recruitment of specific host factors to -1 PRF RNA. Additionally, our findings reveal that while endogenous nucleic acids influence the binding affinity of certain proteins (FUBP3, RPS3A, RPL10A) to -1 PRF RNA, they are not strictly required for these interactions to occur (Fig. 4f).

### SLBP bound to stem loops of SARS-CoV-2 -1 PRF RNA

**Fig. 5:**
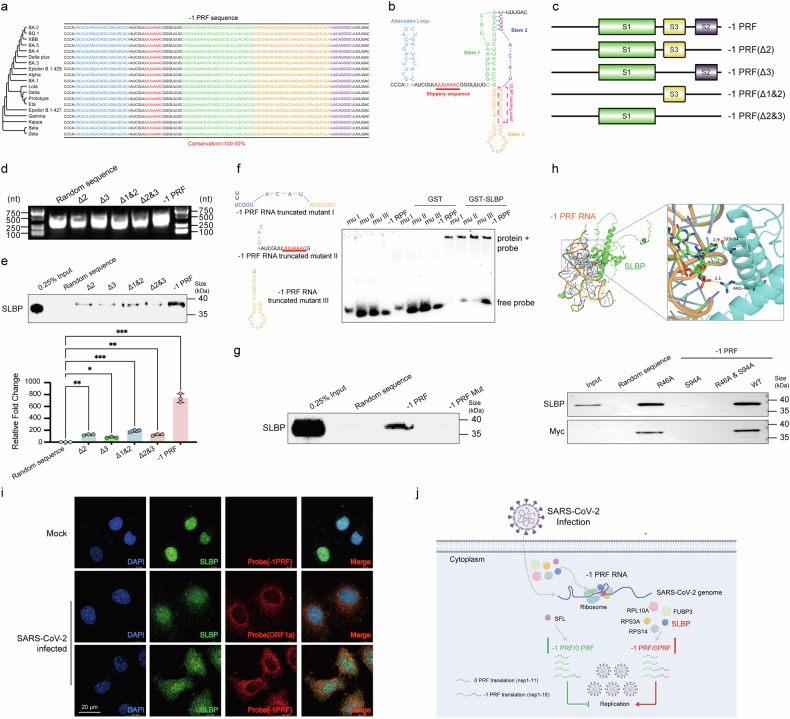
SLBP bound to the stem loop region of SARS-CoV-2 -1 PRF RNA. **a** -1 PRF RNA sequences were conserved among the genome sequences of 19 different SARS-CoV-2 strains. The viral sequences were manually retrieved from the National Center for Biotechnology Information (NCBI). Sequence alignment was conducted using MEGA 11.0 software. **b** The proposed secondary structure of the SARS-CoV-2 -1 PRF element. **c** Schematic representations of the -1 PRF RNA studied, including full -1 PRF RNA, ΔSL2 mutant, ΔSL3 mutant, ΔSL1& 2 mutant, and ΔSL2&3 mutant. The schematic diagram was generated with IBS 2.0 (https://ibs.renlab.org/#/server). **d** RNA probes used were obtained from in vitro transcription. **e** Using cellular proteins extracted from H1299 cells, immunoprecipitation of tRSA-labeled wild-type and mutant -1 PRF RNA probes pulled down SLBP. tRSA-labeled random sequence RNA was used as a negative control (*n* = 3). **f** Interaction between SLBP and wild-type -1 PRF RNA or predicted RNA sequence of -1 PRF RNA using RNA EMSAs. Lane 1, -1 PRF RNA truncated mutant I. Lane 2, -1 PRF RNA truncated mutant II. Lane 3, -1 PRF RNA truncated mutant III. Lane 4, -1 PRF RNA. Lane 5, -1 PRF RNA truncated mutant I + GST. Lane 6, -1 PRF RNA truncated mutant II + GST. Lane 7, -1 PRF RNA truncated mutant III + GST. Lane 8, -1 PRF RNA + GST. Lane 9, -1 PRF RNA truncated mutant I + GST-SLBP. Lane 10, -1 PRF RNA truncated mutant II + GST-SLBP. Lane 11, -1 PRF RNA truncated mutant III + GST-SLBP. Lane 12, -1 PRF RNA + GST-SLBP. Experiments were repeated three times with similar results. **g** Using cellular proteins extracted from H1299 cells, immunoprecipitation of random sequence RNA, wild-type and silent SL3 mutation -1 PRF RNA probes pulled down SLBP. **h** Schematic diagram of interaction models between SLBP and -1 PRF RNA through the molecular docking. The RNA pull-down followed by WB assay was performed to evaluate the interaction between SLBP mutants and -1 PRF RNA. Total cell lysates from H1299 cells transfected with different SLBP variants were used in the experiment. These variants included single mutations where arginine 46 was changed to alanine (R46A), serine 94 was changed to alanine (S94A), and a double mutation (R46A & S94A), where both arginine 46 and serine 94 were replaced with alanine, respectively. Meanwhile, total cell lysates from H1299 cells transfected with wild-type SLBP (WT) were used as a positive control. **i** Co-detection of SLBP with -1 PRF RNA. Polyclonal anti-SLBP was used for SLBP protein immunofluorescence. Scale bar = 20 µm. **j** Schematic diagram of five host factors promoting SARS-CoV-2 replication via regulating frameshifting. SARS-CoV-2 apply the -1 PRF mechanism to switch complete protein translation from ORF1a to ORF1b, while FUBP3, SLBP, RPL10A, RPS3A, and RPS14 bind directly to the SARS-CoV-2 -1 PRF RNA to promote replication via enhancing the switch procedure. The expression of SFL inhibited SARS-CoV-2 replication via reducing frameshifting. The schematic diagram was generated with www.figdraw.com. Data points represent the mean ± SEM. Multiple comparisons were performed using ANOVA with Dunnett’s test. *P < 0.05, **P < 0.01, and ***P < 0.001

Prior research has shown that most RBPs regulate the viral life cycle by binding to specific genomic regions.^29^ We showed that 19 highly transmissible SARS-CoV-2 strains shared the -1 PRF RNA sequence by sequence alignment, including Prototypic (GenBank: NC_045512.2), Alpha (GenBank: MW422255.1), Beta (GenBank: OQ690224.1), BA.1 (GenBank: OL672836.1), BA.2 (GenBank: OM172026.1), BA.3 (GenBank: OV372498.1), BA.4 (GenBank: OW361253.1), BA.5 (GenBank: OW629204.1), BQ.1 (GenBank: OX332805.1), Delta (GenBank: MW931310.1), Delta plus (GenBank: OU315153.1), Epsilon B.1.427 (GenBank: OQ756730.1), Epsilon B.1.429 (GenBank: OQ756586.1), Eta (GenBank: OQ314443.1), Gamma (GenBank: ON507033.1), Kappa (GenBank: OQ204216.1), Lota (GenBank: OQ757092.1), XBB (GenBank: OX343591.1), and Zeta (GenBank: OQ690283.1_P.2) strains, with no mutation site (Fig. 5a). Since SARS-CoV-2 -1 PRF RNA secondary structure forms three stem loops (SLs), identified asSL1, SL2, and SL3 (Fig. 5b)—we hypothesized that the interaction between SLBP and -1 PRF RNA characterized by sequence specificity. To characterize the sequences important for SLBP-mediated effects in depth, we generated four -1 PRF RNA truncation mutants: Δ2, Δ3, Δ1& Δ2, and Δ2& Δ3, with deletions in the predicted SL2, SL3, SL1&SL2, and SL2&SL3 regions, respectively (Fig. 5c, d). Our results indicated SLBP’s affinity for SL1 and SL3 within -1 PRF RNA (Fig. 5e). Additionally, we found that the binding of FUBP3 to the SL1 of -1 PRF RNA, and the binding of RPL10A, RPS3A, and RPS14 to all three SLs, especially SL1 of -1 PRF RNA contributed to their interaction (Supplementary Fig. S4e). Analysis of the findings revealed that RPL10A, RPS3A, and RPS14 exhibit broad interaction capabilities with -1 PRF RNA, engaging at numerous binding sites, while SLBP and FUBP3 showed higher sequence selectivity compared to the three ribosome-related proteins. Overall, the recognition of -1 PRF RNA by SLBP, FUBP3, RPL10A, RPS3A, and RPS14 was sequence-specific.

To further confirm these results, we predicted potential SLBP binding regions on SARS-CoV-2 -1 PRF RNA using the HDOCK web server, focusing on the top 3 predictions. We then performed EMSAs using either biotin-labeled wild-type -1 PRF RNA or truncated -1 PRF RNA probes (including mutants I, II, and III). As shown in Fig. 5f, binding was confirmed in the reverse direction, where GST-SLBP successfully pulled down all three truncated -1 PRF RNA mutants and full-length -1 PRF RNA. The results indicated that GST-SLBP had stronger binding affinity for truncated mutant I, which contains SL2 and SL3. To further identify the main binding region of SLBP, we introduced a silent mutation in SL3 as previously described^30^ and performed RNA pull-down assays. Disruption of SL3 in the pseudoknots strongly suppressed the interaction between -1 PRF RNA and SLBP (Fig. 5g). Together, our RNA pull-down and EMSA analyses suggest that SL3 is likely the main SLBP binding region. Moreover, the SLBP binding sequence reported was 26 nucleotide, CCAAAGGCUCUUUUCAGAGCCACCCA.^31,32^ Thus, we can infer from these data that AUGUCGUA is the primary RNA binding motif of SLBP.

To assess the binding interactions and molecular docking patterns between -1 PRF RNA and SLBP, we employed the Uniprot and HDOCK web servers for computational analysis. The resulting 3D models of the protein-RNA complexes were then rendered and examined using PyMOL visualization software (Fig. 5h). The amino acids R46 and S94 in the SLBP protein interacted with base 104 (A) in -1 PRF RNA (Fig. 5h). We established Myc-SLBP overexpressing H1299 cells containing single point mutations (R46A or S94A) and a double mutation (R46A/S94A). Further validation through RNA pull-down WB assays demonstrated that the S94 amino acid of SLBP is essential for its binding to -1 PRF RNA (Fig. 5h). Western blot analysis confirmed equivalent expression of all mutant proteins (Supplementary Fig. S7), demonstrating that the defective RNA binding of S94A is due to the loss of serine-94 function rather than protein instability. Sequence analysis revealed high conservation in the RNA binding domain (RBD) across all three human SLBP isoforms (Supplementary Fig. S8). In this work, we specifically studied isoform 3, which was identified as the predominant form when cloning SLBP coding sequence from cells. Structural mapping confirmed that the S94 was located in the conservative RBD region (Supplementary Fig. S8). We performed single-molecule fluorescence in situ hybridization (smFISH) assays utilizing fluorescently tagged probes targeting viral ORF1a and -1 PRF RNA, which showed that SLBP protein co-localized with ORF1a and -1 PRF RNA (Fig. 5i). Additionally, FUBP3 protein co-localized with ORF1a and -1 PRF RNA, with ZAP-S serving as a positive control (Supplementary Fig. S9a). We also demonstrated that three ribosomal proteins—RPL10A, RPS3A, and RPS14—co-localize with -1 PRF RNA following SARS-CoV-2 infection (Supplementary Fig. S9b). This experimental evidence supports the association between these specific ribosomal proteins and the -1 PRF RNA during viral infection, providing direct visualization of their interaction in situ. Overall, our results indicate a recruitment mechanism of SLBP with sequence specificity, further supporting the sequence-specific interaction between SLBP and SARS-CoV-2 -1 PRF RNA.

## Discussion

The fast creation of new SARS-CoV-2 mutant strains heightens the likelihood of variations developing treatment resistance and evading immunological responses.^33^ While vaccinations, such as BNT162b2 and CoronaVac, are crucial for mitigating the risk of severe COVID-19, they do not entirely prevent infection by epidemic strains.^34^ The production of viral RNA-dependent RNA polymerase and subsequent nonstructural proteins of SARS-CoV-2 relies on -1 PRF, a critical event in the translation of the SARS-CoV-2 RNA genome.^35^ The -1 PRF RNA element exhibits strong conservation across SARS-CoV-2 variants. Moreover, the sequence shares greater than 90% homology between SARS-CoV and SARS-CoV-2. Specifically, there are 6 base substitutions in the 30 bases of the attenuator loop sequence (80.00% homology) and only 1 substitution in the remaining 89 bases of the -1 FSE sequence (98.88% homology).^16^ As previously noted, -1 PRF RNA has been considered a potential breakthrough target for treating COVID-19.^10^ To date, only a few host factors, including SFL,^18^ ZAP-S,^19^ and eIF2A,^20^ have been identified as participants in the regulating SARS-CoV-2 programmed -1 ribosomal frameshifting. Thus, many important host factors involved in frameshift regulation during COVID-19 development have yet to be thoroughly clarified. In this study, using -1 FSE and -1 PRF RNA as baits, we identified several *trans*-acting host factors that influence SARS-CoV-2 frameshifts. As expected, multiple ribosome-related proteins were found to be engaged in this process. Our study elucidates the mechanism by which SLBP interaction with -1 PRF RNA promotes SARS-CoV-2 frameshifting and enhances viral replication.

While viral protein translation generally depends on multiple host factors, the cooperative mechanisms of specific host factor combinations remain poorly understood. Previous studies on PRRSV frameshift regulation demonstrated that a triple frameshift stimulator complex forms for frameshifting, comprising NSP1β, human PCBP, and ssRNA containing the slippery sequence and C-rich motif.^36^ However, to our knowledge, the frameshifting regulatory complex has not been documented in SARS-CoV-2. Our results showed that SLBP, FUBP3, RPL10A, RPS3A, and RPS14 regulate SARS-CoV-2 -1 PRF via heterogeneous RNA-protein complexes, likely modulating viral translation dynamically. Biochemical characterization revealed these factors assemble into multiple ribonucleoprotein (RNP) complexes with molecular weights spanning from ~93 kDa to >670 kDa, as demonstrated by native PAGE and immunoblotting analyses. The observed heterogeneity in complex sizes suggests these host factors may function through dynamic, multi-component regulatory modules to modulate -1 PRF efficiency. Additional structural and functional analyses are needed to comprehensively clarify the composition and mechanistic contributions of these complex assemblies to viral frameshifting regulation.

Like all viruses, SARS-CoV-2 must utilize host ribosomes to complete viral protein translation and replication due to its lack of translation machinery. While ribosome-related proteins would theoretically be expected to participate in frameshifting regulation, no studies have confirmed this to date. In our study, we specifically characterized three ribosome-related proteins (RPL10A, RPS3A, and RPS14) and two nonribosome-related proteins (FUBP3 and SLBP) involved in SARS-CoV-2 frameshift. Notably, FUBP3 and RPS14 were also identified as prevalent targets in genome-wide screens for proteins interacting with SARS-CoV-2 RNA.^37^ We investigated the role of these five host factors in viral replication. Loss of these factors through siRNA targeting inhibited SARS-CoV-2 replication, while their overexpression promoted viral replication. Furthermore, we utilized our developed and well-characterized luciferase-based SARS-CoV-2 -1 PRF reporters, demonstrating that their proviral activity functions by promoting frameshifting. Building on the work of PrismNet prediction, our studies reported here focus on SLBP. While prior reports indicate that SLBP depletion elevates HIV-1 unspliced RNA levels^27^ and impedes the effective production of infectious HCMV virions,^25^ the influence of SLBP on cellular responses under SARS-CoV-2 infection has not been previously studied. Quantitative analysis revealed a clear divergence in SLBP’s regulatory influence: siRNA-induced SLBP knockdown reduced viral replication by approximately 55%, whereas overexpression enhanced replication by only approximately 5%, indicating its predominant function as a replication-permissive factor. These results suggest SLBP functions as a pivotal scaffold protein that modulates RNA-protein interactions, with its depletion reducing FUBP3/RPS3A binding while promoting RPL10A association with SARS-CoV-2 -1 PRF RNA. Additionally, we employed RNA Pull-Down and EMSA experiments to demonstrate that SLBP primarily binds to the -1 PRF RNA sequence AUGUCGUA, and further confirmed that this interaction depends on the amino acid residue S94. These findings suggest that investigating SLBP inhibitors as potential suppressors of SARS-CoV-2 replication could be a promising direction for future research.

Shiftless (*SFL*) is the first recognized interferon stimulating gene (*ISG*) that can inhibit -1 PRF and target the translation recoding process,^38,39^ herein named interferon regulated antiviral (*IRAV*) gene, resulting in the restriction of the replication of multiple viruses,^39–41^ including SARS-CoV-2.^10^ The interaction between SFL and SARS-CoV-2 RNA was reported.^37^ However, whether there is an interaction between SFL and SARS-CoV-2 -1 PRF RNA currently remains unknown. Through an RNA pull-down experiment, we confirmed the interaction between SFL and SARS-CoV-2 -1 PRF RNA utilizing H1299 total cell lysate (Supplementary Fig. S4e). Additionally, we performed RNA‒protein pull-down and protein‒protein pull-down assays following overlapping analysis. We found that all 5 proteins, including FUBP3, SLBP, RPL10A, RPS3A, and RPS14, were in the overlapping list (Supplementary Fig. S4f, Data set 2). Moreover, five proteins above were identified to interact with SFL and -1 PRF RNA using a pull-down assay (Supplementary Fig. S4g). Of interest, although five host factors and SFL played contrary roles in SARS-CoV-2 frameshifts, experimental evidence supported that there were interactions between SFL and these proteins. Motivated by our screening results, we set out to investigate their interactions. Our results supported that an interaction network was formed among these host factors. Furthermore, we confirmed that the wild-type SFL and SFL truncated mutant (164-199) maintained their frameshifting restriction function (Supplementary Fig. S10a–e).

In conclusion, our study identifies key host factors—including ribosome-associated proteins (RPL10A, RPS3A, RPS14) and RNA-binding proteins (SLBP, FUBP3)—that dynamically regulate SARS-CoV-2 -1 PRF through heterogeneous RNP complexes. SLBP, in particular, acts as a scaffold protein, with its depletion altering the binding affinities of the specific factors to -1 PRF RNA and significantly impairing viral replication. Notably, these host factors interact with the SFL, suggesting a complex regulatory network balancing proviral and antiviral effects. Structural and functional analyses reveal that SLBP binds the -1 PRF motif AUGUCGUA via residue S94, offering a potential target for inhibition. These findings advance our understanding of host-mediated frameshift regulation and propose novel therapeutic avenues, such as SLBP inhibitors, to disrupt SARS-CoV-2 replication. The development of therapeutics targeting SLBP, particularly through structure-guided approaches like molecular docking, offers substantial potential for combating SARS-CoV-2 and related coronaviruses. Future studies should explore the compositional dynamics of these RNP complexes and their interplay with innate immune responses.

## Materials and methods

### Cells

H1299, HEK293T, and Huh7 cell lines were purchased from ATCC (Manassas, VA, USA). H1299 cells were maintained in RPMI-1640 (Gibco) with 10% Fetal Bovine Serum (FBS) (Gibco), 100 μg/ml streptomycin and 100 U/ml penicillin. HEK293T cells were grown in DMEM (Gibco) containing 10% FBS, and Huh7 cells were cultured in MEM (Gibco) with 10% FBS.

### Plasmid construction

Fragments of random sequences, -1 FSE, -1 PRF, -1 PRF (Δ2), -1 PRF (Δ3), -1 PRF (Δ1& Δ2), and -1 PRF (Δ2& Δ3), were synthesized (Tsingke Biotechnology Co., Ltd., Beijing, China) and inserted into the pcDNA3.0-tRSA (the tRNA scaffold to a streptavidin aptamer) vector (a gift from Professor Lingling Chen) with the T7 promoter. Human *SFL* isoform 1 (*SFL*; NM_018381.3) and shorter isoform 2 (*SFLS*; NM_001308277.1) coding regions were subcloned into pcDNA4.0, generating C-terminal Flag-tagged constructs. To generate the dual fluorescence reporter constructs pHRF-FSE(-1) and pCIG-mCherry-FSE(-1)-EGFP, the frameshift sequence of SARS-CoV-2 was interposed either Renilla and Firefly luciferase genes or mCherry and EGFP coding sequences. For constructs pHRF-FSE(0) and pCIG-mCherry-FSE(0)-EGFP, the SARS-CoV-2 frameshift mutation, including a silent variant, was incorporated. The FSE 0 plasmid we designed truncated the signal post-Renilla luciferase translation, with no Firefly luciferase expression. For FSE -1 plasmid, the upstream ORF encodes Renilla luciferase with a conventional ATG start codon in the standard reading frame. Meanwhile, expression of the downstream Firefly luciferase ORF—which lacks its own initiation codon—relies entirely on SARS-CoV-2’s -1 PRF mechanism to shift translation into the alternate reading frame. The frameshifting efficiency was determined using a dual-luciferase reporter system. The ratio of Firefly to Renilla luciferase activity (F/R) was calculated by dividing the Firefly/Renilla ratio of the test sample by that of the in-frame control. F/R was calculated using the following formula: Firefly luciferase luminescence value/Renilla luciferase luminescence value (F/R) = (Firefly_test_/Renilla_test_)/(Firefly_control_/Renilla_control_). In the control construct, both luciferases are produced in equal amounts. The results were obtained from at least three independent experimental replicates. The coding sequences of human *FUBP3* (NM_003934.2), *SLBP* (NM_001306075), *RPL10A* (NM_007104), *RPS3A* (NM_001006.5), and *RPS14* (NM_005617.4) were cloned into pcDNA4.0 with a Myc-tag at the C-terminus. Site-directed mutagenesis was performed using the Mut Express II Fast Mutagenesis Kit V2 (Vazyme, C214) according to the manufacturer’s instructions. Plasmids were transfected utilizing TransIT®-LT1 Transfection Reagent (MIR2300, Mirus). The *SLBP* gene was inserted into a bacterial expression vector (pGEX-6P-1), tagged with GST. To facilitate the in vitro translation study, a pcDNA4.0-based frameshift reporter vector for SARS-CoV-2 was constructed. The vector included the T7 promoter, β-globin 5′ and 3′ UTRs and a 34 nucleotide poly-(A) tail. The viral insert spanned nucleotides 12686 through 14190 (reference strain NC_045512), with an N-terminal Flag-tag incorporated for subsequent protein detection purposes.

The primers used are listed in Supplementary Table S1.

### RNA interference (RNAi)

*FUBP3* siRNA, *SLBP* siRNA, *RPL10A* siRNA, *RPS3A* siRNA, *RPS14* siRNA, and negative control siRNA were acquired from Gene Pharma and utilized at a final concentration of 50 nmol/L. GP-transfect-Mate (211010, Gene Pharma Co., Ltd., China) was utilized for transfection according to the manufacturer’s guidelines. The corresponding sense and antisense sequences for each siRNA are listed in Supplementary Table S2.

### RNA pull-down assay

To generate RNA pull-down probes, pcDNA3.0-random sequence-tRSA, pcDNA3.0-(-1 FSE)-tRSA, pcDNA3.0-(-1 PRF)-tRSA, pcDNA3.0-(-1 PRF Δ2)-tRSA, pcDNA3.0-(-1 PRF Δ3)-tRSA, pcDNA3.0-(-1 PRF Δ1& Δ2)-tRSA, pcDNA3.0-(-1 PRF Δ2& Δ3)-tRSA, and pcDNA3.0-(-1 PRF Mutation)-tRSA were linearized by EcoRV (#R3195L, NEB) digestion, followed by in vitro transcription utilizing T7 RiboMAX™ Large Scale RNA Production Systems (P1300, Promega). Then, identical quantities of the samples were incubated with BeyoMag™ Streptavidin Magnetic Beads (P2151, Beyotime) for 6 h at 4 °C. The enrichment of proteins bound by probes was examined.

The sequence of tRSA tag was as follow:

5′-GGAATTGAAAAAAAAAAAAGCCCGGATAGCTCAGTCGGTAGAGCAGCGGCCTCGACCAGAATCATGCAAGTGCGTAAGATAGTCGCGGGTCGAGGCCGCGTCCAGGGTTCAAGTCCCTGTTCGGGCGCCACTGCAGAAAAAAAAAAAAGAATTC-3′

The specific RNA probes used for each sample are described in Supplementary Table S3.

### Western blotting

Cell lysates were prepared using RIPA buffer (P0013B, Beyotime, Shanghai, China) with freshly added protease inhibitor cocktail (G2006, Servicebio, Wuhan, China) and phosphatase inhibitor (G2007, Servicebio, Wuhan, China) for 30 min on ice. Protein samples combined with loading buffer were denatured at 95 °C and promptly introduced into a 10% gel and resolved by SDS‒PAGE. Following SDS-PAGE, proteins underwent transfer onto a PVDF membrane (#1620177, Bio-Rad). Antibody details are in Supplementary Table S4.

### Coimmunoprecipitation assay

For co-IP assays, equal amounts of cell lysate were treated overnight at 4 °C with anti-Flag Affinity Gel (P2282, Beyotime, Shanghai, China) or anti-Myc magnetic beads (P2118, Beyotime, Shanghai, China). Subsequently, the gels or beads were washed five times with cell lysis buffer, and the immunoprecipitated complexes were suspended in 2 × SDS loading buffer, boiled, and analyzed via SDS‒PAGE for Western blotting.

### SARS-CoV-2 infection

The virus strains used in this study were the prototypic SARS-CoV-2 strain (GD108 from Guangdong Center for Disease Control and Prevention (CDC)), Omicron variant (B.1.1.529 CCPM-B-V-049-2112-18 from the Institute of Laboratory Animal Sciences, Chinese Academy of Medical Sciences and Peking Union Medical College), and HCoV-229E strain (from the Institute of Medicinal Biotechnology, Chinese Academy of Medical Sciences and Peking Union Medical College). The viruses were propagated in Vero-E6 cells cultured in DMEM supplemented with 10% FBS and 1% penicillin‒streptomycin-amphotericin B solution (Solarbio, P7630). The viral titration for infection was quantified using the plaque assay. To study the effects of the indicated proteins on SARS-CoV-2 infection, Huh7 cells were employed. Cells were infected with SARS-CoV-2 at an MOI of 0.05. All work with SARS-CoV-2 was performed in a biosafety level (BSL) 3 laboratory following approved standard operating procedures.

### RNA extraction and quantitative reverse transcription-polymerase chain reaction

RNA isolation was performed with the Direct-zol™ RNA MiniPrep kit (R2052, Zymo) following the manufacturer’s protocol. The extracted RNA was subjected to one-step RT‒qPCR with the specific primers and probes (Supplementary Table S1). The 10 μl reaction mixtures were set up with 2.5 μl of RNA. The cycling parameters were: 2 min at 25 °C, 15 min at 50 °C, 2 min at 95 °C; then 40 cycles of 5 s at 95 °C and 31 s at 58 °C.

### Immunofluorescence staining

Incubated cells were fixed with 4% paraformaldehyde and permeabilized using immunostaining permeabilization buffer containing Triton X-100 (P0096, Beyotime). Afterward, cells were blocked with 1% (w/v) bovine serum albumin in PBS-T (0.1% [v/v] Tween 20) and incubated with primary and secondary antibody (Supplementary Table S4). Additionally, the smFISH probes were labeled with Cy3. Nuclei were dyed with Fluoroshield^TM^ with DAPI (Sigma, SLCB0123). Slices were visualized using a Leica SP8 confocal laser scanning microscope.

### Purification of recombinant proteins

The N-terminal GST-tagged recombinant SLBP was expressed in *E. coli Rosetta* cells through overnight induction with 0.1 mM IPTG at 16 °C. Cells were lysed using Bacterial Active Protein Extraction Reagent (P0013Q, Beyotime, Shanghai, China). The lysate was cleared by centrifugation and SLBP was isolated utilizing Glutathione Sepharose^TM^ 4 Fast Flow (17-5132-01, GE Healthcare, SWEDEN). Then, elution buffer (pH 7.9–8.1) for GST-Sefinose (TM) resin (C600325-0500, Sangon Biotech, Shanghai, China) was used to elute GST-tagged proteins. Purified SLBP was promptly aliquoted and kept at −80 °C.

### In vitro translation

The pcDNA4.0 SARS-CoV-2 frameshift reporter vector was linearized by XhoI (#R0146L, NEB) as the DNA template. The linearized DNA template was transcribed and translated with the TNT® T7 Coupled Reticulocyte Lysate System (L4610, Promega). Typical reactions were comprised of 12.5 μl TNT® Lysate, 1 μl TNT® Reaction Buffer, 0.5 μl TNT® RNA Polymerase, 0.25 μl Amino Acid Mixture Minus Leucine, 0.25 μl Amino Acid Mixture Minus Methionine, 0.5 μl RNasin® Ribonuclease Inhibitor, 400 ng linearized DNA template, GST-SLBP, and Nuclease Free Water to a final volume of 25 μl. Reactions were incubated for 1 h at 30 °C. Samples were added to 75 μl of 2 × SDS‒PAGE loading buffer and boiled for 10 min at 95 °C. The products were resolved on FuturePAGE^TM^ 4–12% 15-well gels (ET15412Gel, ACE Biotech, Nanjing, China) and transferred onto PVDF membranes (#1620177, Bio-Rad).

### SLBP binding site prediction based on PrismNet

SLBP binding was anticipated utilizing PrismNet (http://prismnetweb.zhanglab.net/) as previously delineated by Professor Lei Sun,^42^ a deep learning model founded on RNA sequence and in vivo RNA structural data. If the binding probability result from the PrismNet model exceeded 0.85, we designated the sequence window as a potential binding site for SLBP.

### Electrophoretic mobility shift assays

Biotin-tagged RNA probes were synthesized with the Pierce™ RNA 3′ End Biotinylation Kit (20160, Thermo). Labeled RNA (40 pmol) was incubated with purified GST-SLBP (4.5 µg) in EMSA/Gel-Shift Binding Buffer (GS005, Beyotime) for 20 min at 25 °C. Subsequently, samples were swiftly applied to 6.5% acrylamide nondenaturing gels, which were electrophoresed at 100 V for 30 min in an ice bath utilizing 0.5 × TBE as the running buffer until free and bound RNA species were separated, and transferred onto positively charged nylon membranes (FFN10, Beyotime). After the UV-crosslinking procedure, the Chemiluminescent EMSA Kit (GS009, Beyotime) was used for the shift assay according to the manufacturers’ instructions. The RNA probe sequences are listed in Supplementary Table S3.

### Single-molecule fluorescence in situ hybridization

Briefly, Huh7 cells were grown on coverslips in a 12-well plate and infected with SARS-CoV-2 (MOI 0.01) for 48 h before fixation with 4% paraformaldehyde. Coverslips were sterilized in 80% and stored in 100% ethanol. Cells were permeabilized with PBS-T/0.1% Triton X-100, washed with PBS-T, then pre-hybridized twice for 20 min each at 37 °C in wash buffer (2 × SSC, 10% formamide, plus RNase inhibitor in the reaction). Hybridization was conducted in hybridization solution (2 × SSC, 10% formamide, 10% dextran sulphate, 1 mg/ml tRNA, 0.2 mg/ml BSA, plus RNase inhibitor in the reaction) using 1 µM smFISH probes overnight at 37 °C. Subsequent to the overnight hybridization, cells were subjected to three 15-min washes in wash buffer at 37 °C followed by washing for 10 min in 1 × SSC. The cells were subsequently blocked with PBS supplemented with 3% FBS, 0.5% Triton X-100, plus RNase inhibitor for 1 h at room temperature, followed by an overnight soak with the anti-SLBP antibody (ab221166, Abcam) at 4 °C. After thorough washing with PBS-T, the samples were treated with the iFluor™ 488-conjugated goat anti-rabbit IgG polyclonal antibody for an additional hour, followed by another round of PBS-T washes. Nuclei were then counterstained with DAPI. Images were captured using a Leica TCS SP8 microscope (Germany). The smFISH probe sequences employed in this experiment, adapted from a prior study with slight adjustments,^43^ are detailed in Supplementary Table S3.

### Naive polyacrylamide gel electrophoresis analysis

The -1 PRF RNA pull down products in this study were all conducted in the same manner. A 20 μL samples was resolved using a NativePAGE™ Novex® 4–16% Bis-Tris gel with Native-PAGE 1x Runing Buffer. Native-PAGE gels were transferred to PVDF and immunodetected.

### Statistical analyses

All statistical analyses were performed utilizing GraphPad Prism software (version 8.0, GraphPad Software, CA, USA). Multiple group comparisons were conducted using one-way analysis of variance (ANOVA) accompanied by Dunnett’s post hoc tests. All data are presented as the means ± SDs indicated in the figure legends, and at least three biologically independent experiments were performed. Statistical significance was set at *P < 0.05, **P < 0.01, ***P < 0.001; “ns” denoted non-significance.

## Supplementary information

Supplementary Materials

Data set1

Data set2

## Source data

Source data

## Data Availability

The authors confirm that all data supporting this study’s conclusions are included in the manuscript. The proteomics data obtained through mass spectrometry have been archived in the ProteomeXchange Consortium’s database, accessible via the iProX repository under accession number PXD042504 (https://www.iprox.cn//page/SCV017.html?query=PXD042504).
