# Supplementary material for: Stem loop binding protein promotes SARS-CoV-2 replication via -1 programmed ribosomal frameshifting
Source: Signal Transduct Target Ther. 2025 Jun 13;10:192. doi: 10.1038/s41392-025-02277-w (PMC12166091; doi:10.1038/s41392-025-02277-w)
Supplement: Supplementary file 1 — Supplementary Materials [file 41392_2025_2277_MOESM1_ESM.docx]

Supplementary Materials for

Stem loop binding protein promotes SARS-CoV-2 replication

via -1 programmed ribosomal frameshifting

Tanxiu Chen^*^, Ruimin Zhu^*^, Tingfu Du^*^, Hao Yang, Xintian Zhang, Zhixing Wang, Yong Zhang, Wenqi Quan, Bin Yin, Yunpeng Liu^#^, Shuaiyao Lu^#^, Xiaozhong Peng^#^

Correspondence to: [pengxiaozhong@pumc.edu.cn](mailto:pengxiaozhong@pumc.edu.cn)

**This PDF file includes:**

Figures. S1 to S10

Tables S1 to S4

**Other Supplementary Materials for this manuscript include the following:**

None.


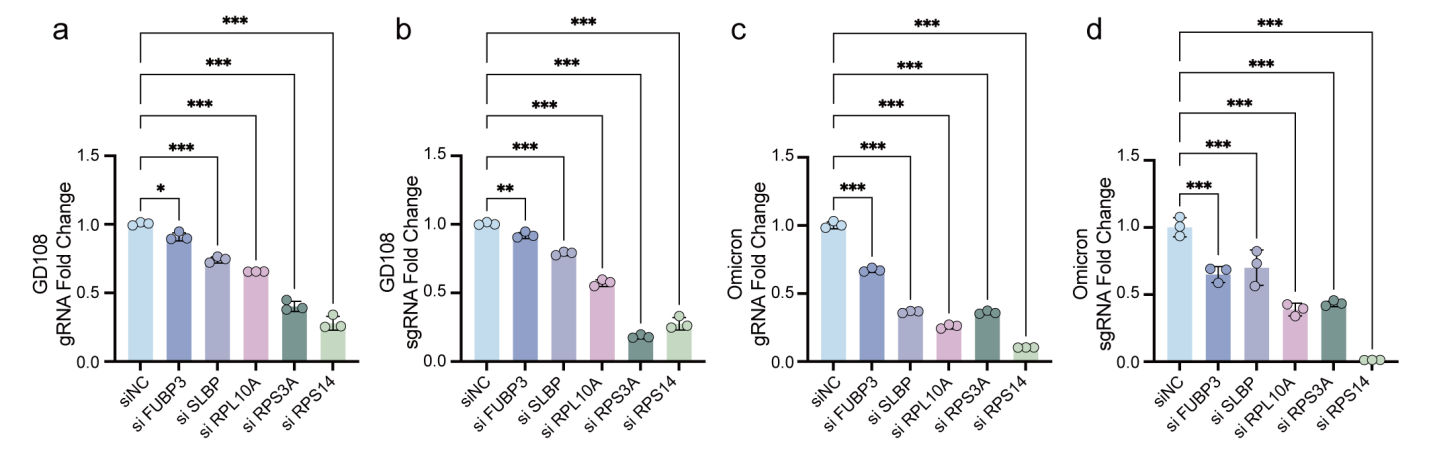


**Supplementary Figure S1. Knockdown of FUBP3, SLBP, RPL10A, RPS3A, and RPS14 inhibited SARS-CoV-2 replication in H1299 cells.**

**a, b** Knockdown of host factors decreased the expression levels of gRNA and sgRNA of SARS-CoV-2 in H1299 cells infected with the GD108 strain (MOI=0.05, 48 h). **c, d** Knockdown of host factors decreased the expression levels of gRNA and sgRNA of SARS-CoV-2 in H1299 cells infected with the Omicron strain (MOI=0.05, 48 h) (a-d, n=3, *P < 0.05, **P < 0.01, and ***P < 0.001). Data points represent the mean±SEM. Multiple comparisons were performed using ANOVA with Dunnett’s test.


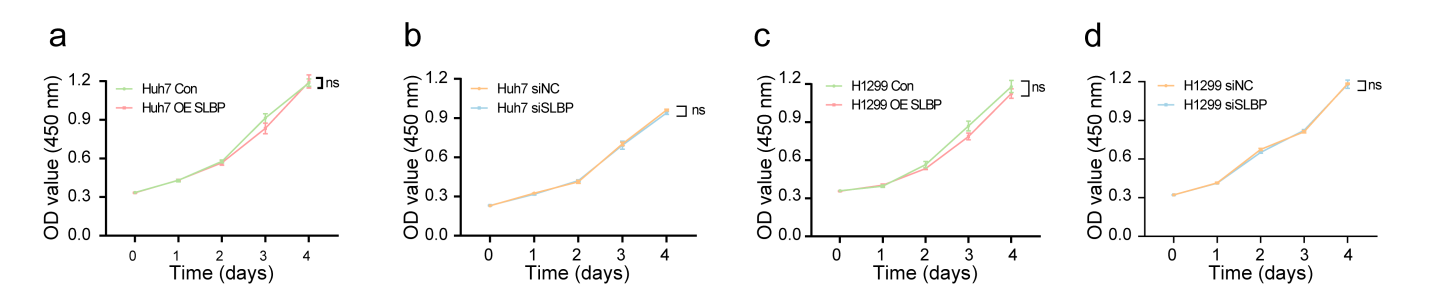


**Supplementary Figure S2. The effects of SLBP on Huh7 and H1299 cells proliferation.** (a-d, n=3, ns, not significant)

**
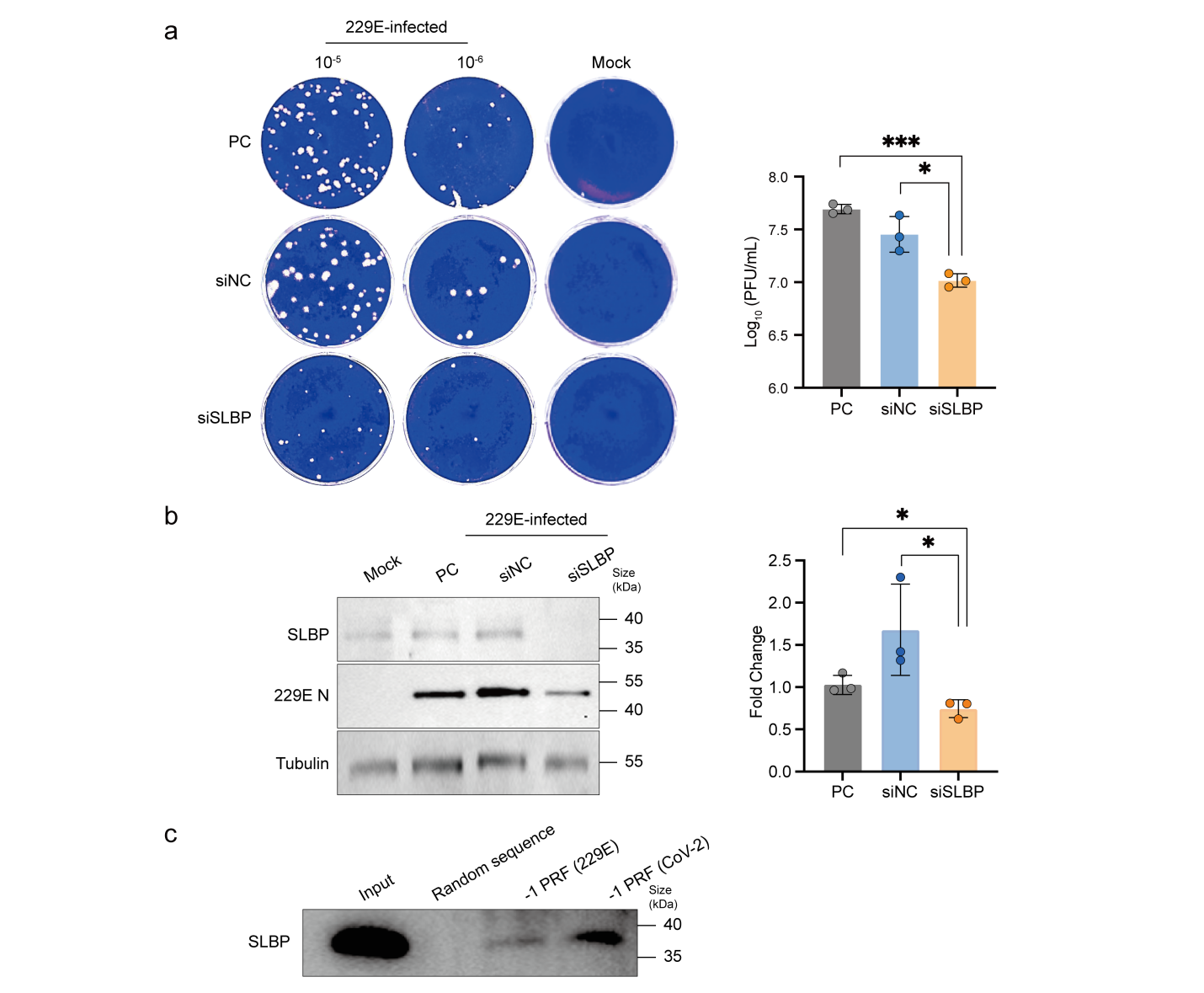
**

**Supplementary Figure S3. The effects of SLBP on 229E replication were determined by knocking down SLBP performing plaque and WB analysis.**

**a** Quantitative measurement of the plaque number on Vero cells treated with cell culture supernatant from 229E−uninfected or 229E−infected (MOI=0.2) Huh7 cells. Vero cells were overlaid with agar, and stained with crystal violet at 5 dpi. The column graph showed the 229E titer in the PFU/mL unit that was counted by PFU assay. **b** Knockdown of SLBP decreased the N protein expression level in Huh7 cells infected with 229E (a-b, n=3, *P < 0.05, and ***P < 0.001). **c** The SLBP protein was interacted with 229E -1 PRF RNA, and SARS-CoV-2 -1 PRF RNA was seted as positive control using RNA pull down assay. Data are the mean±SEM. Multiple comparisons were performed using ANOVA with Dunnett’s test.


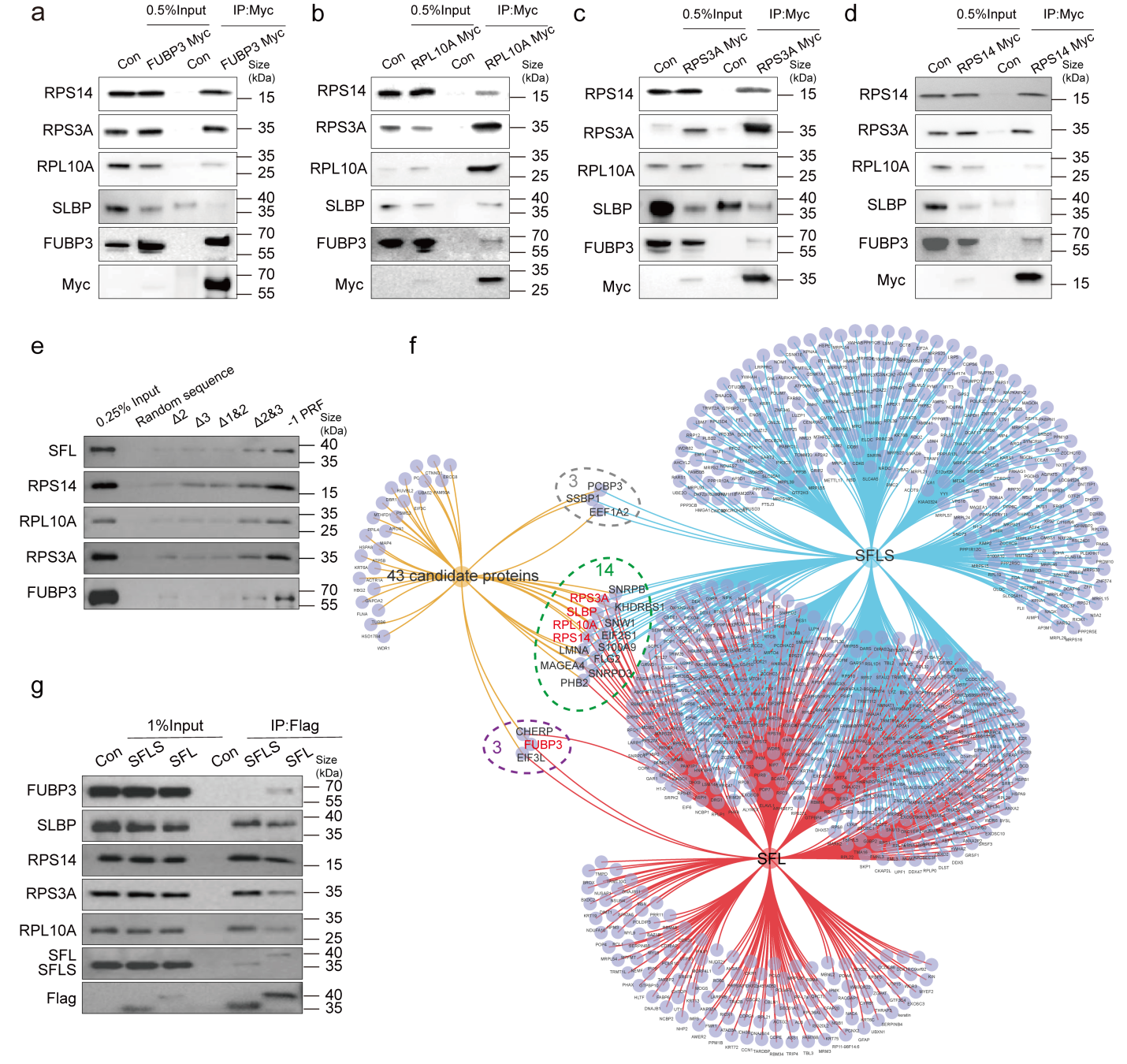


**Supplementary Figure S4. An interaction network among several SARS-CoV-2 -1 PRF RNA-bound host factors.**

**a–d** The coimmunoprecipitation (co-IP) assay confirmed the interaction among SLBP, FUBP3, RPL10A, RPS3A, and RPS14. **e** A RNA pull-down assay followed by western blot analysis was performed to determine the interaction between SARS-CoV-2 -1 PRF RNA or truncated mutants and five host factors, including FUBP3, RPL10A, RPS3A, RPS14, and SFL, using H1299 cell lysates. **f** The overlapping cointeraction proteins among -1 FSE RNA, -1 PRF RNA, SFL, and SFLS were identified using Venn diagram analysis. **g** A Co-IP assay was performed to analyze the proteins interacting with SFL and SFLS using H1299 cells. Experiments were repeated three times with similar results.


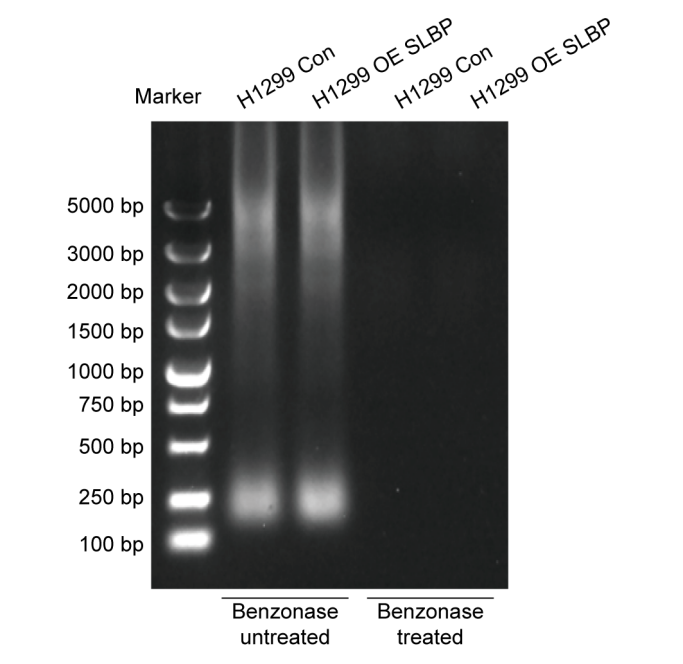


**Supplementary Figure S5. Nucleic acid gel electrophoresis to verify nucleic acid elimination from whole-cell lysate.**

**
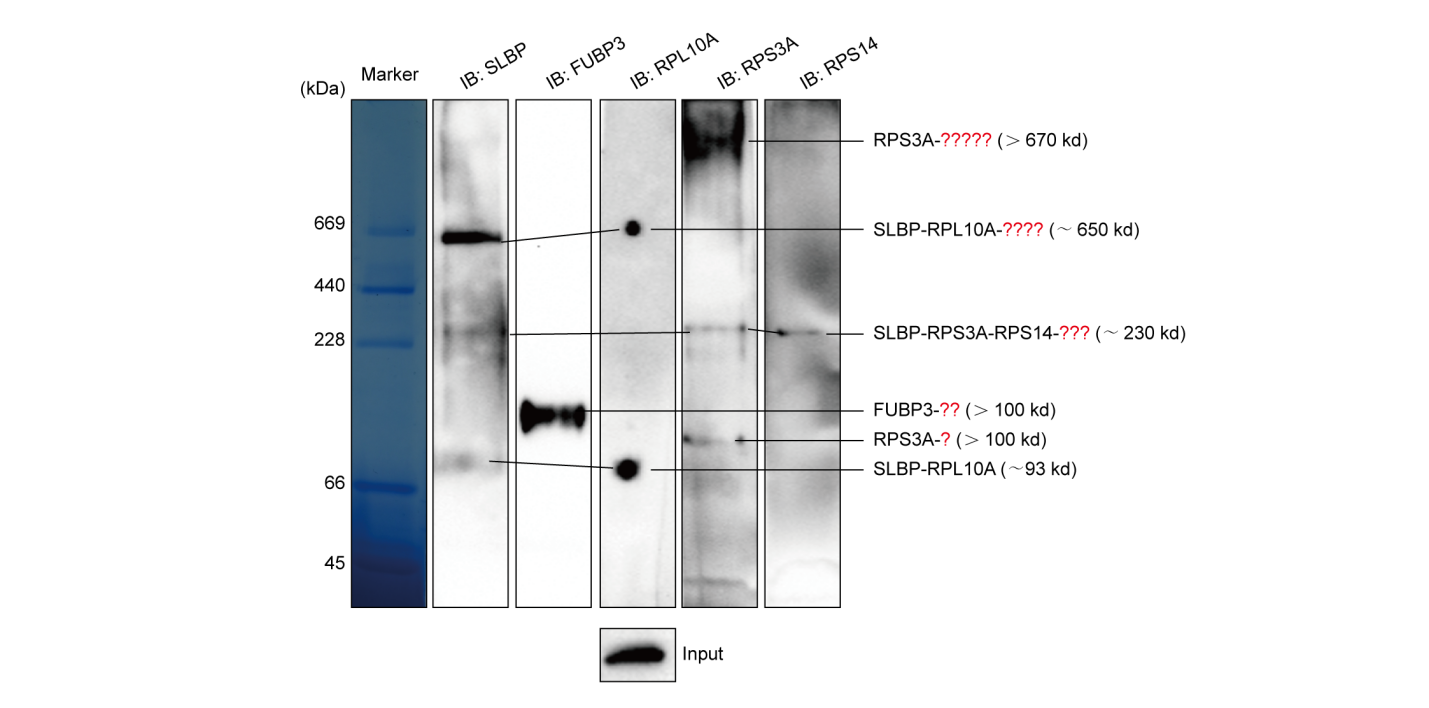
**

**Supplementary Figure S6. Native-PAGE Analysis and Immunoblotting of -1 PRF RNA Pull Down Complex.**

Top panel: Immunoblot detection of the -1 PRF RNA pull-down complex using antibodies against SLBP, FUBP3, RPL10A, RPS3A, and RPS14. Bottom panel: Western blot analysis of RPL10A in input whole-cell lysates from RNA pull-down experiments (H1299 cells).


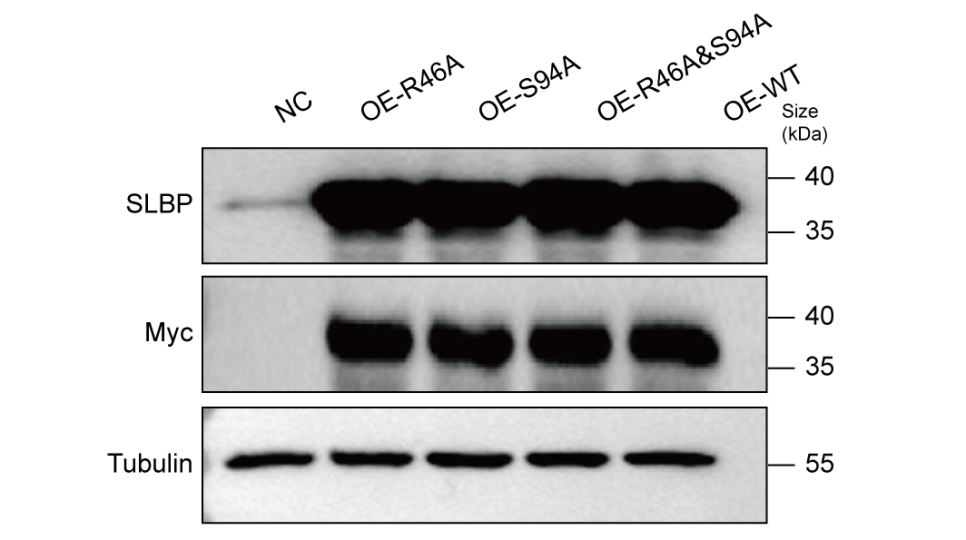


**Supplementary Fig. S7 Detection of SLBP Mutants in H1299 Cells by Immunoblotting.**

Cell lysates from the following H1299 cell lines were analyzed by immunoblotting: Empty vector control (NC); Myc-tagged SLBP-R46A mutant (OE-R46A); Myc-tagged SLBP-S94A mutant (OE-S94A); Myc-tagged SLBP-R46A&S94A (OE-R46A&S94A); and Myc-tagged wild-type SLBP (OE-WT). The expression levels of the R46A, S94A, R46A&S94A SLBP mutants, and wild-type SLBP in H1299 cells were comparable.


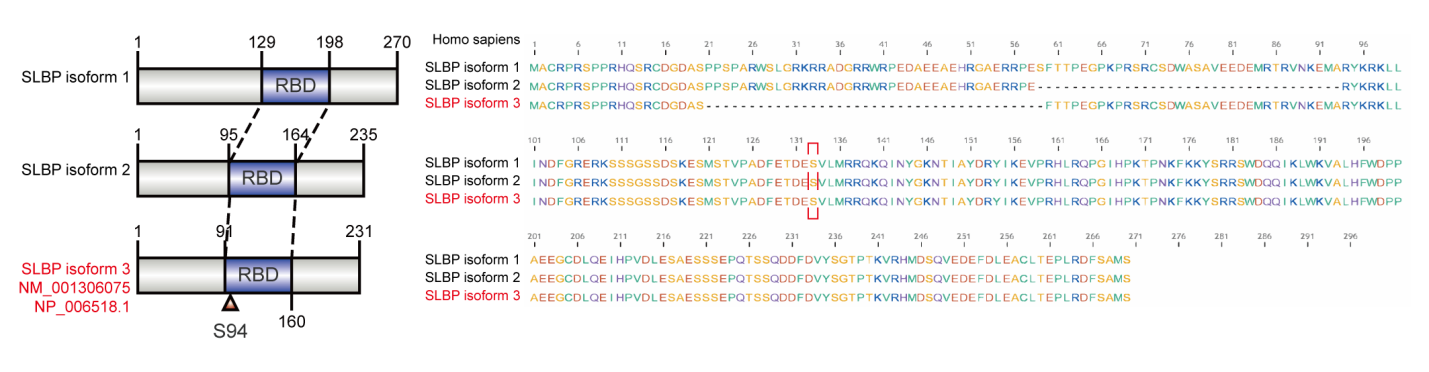


**Supplementary Fig S8.** **Schematic showing the domain organization and sequence alignment of three human SLBP isoforms.**

The RNA binding domain is designated the “RBD”. The -1 PRF RNA binding site S94 have been indicated. The schematic diagram was generated with [IBS 2.0 (https://ibs.renlab.org/#/server).](http://www.figdraw.com.)


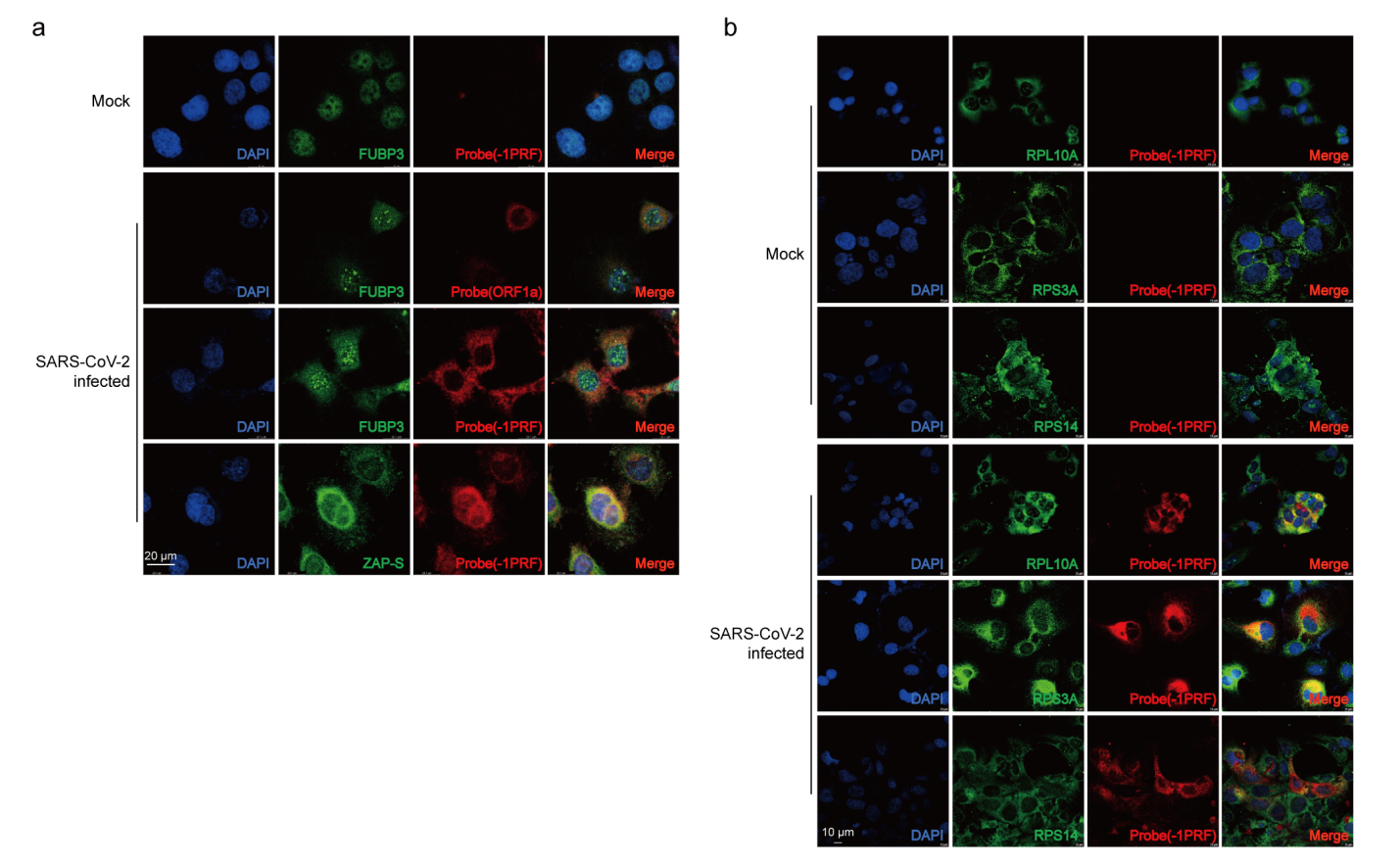


**Supplementary Figure S9.** **Subcellular colocalization of FUBP3 and ribosomal proteins with -1 PRF RNA by confocal microscopy.**

**a** The FUBP3 protein was located with ORF1a and -1 PRF RNA, and ZAP-S was seted as positive control using laser confocal microscopy. Scale bar = 20 µm. **b** The RPL10A, RPS3A, and RPS14 protein were located with -1 PRF RNA using laser confocal microscopy. Scale bar = 10 µm.


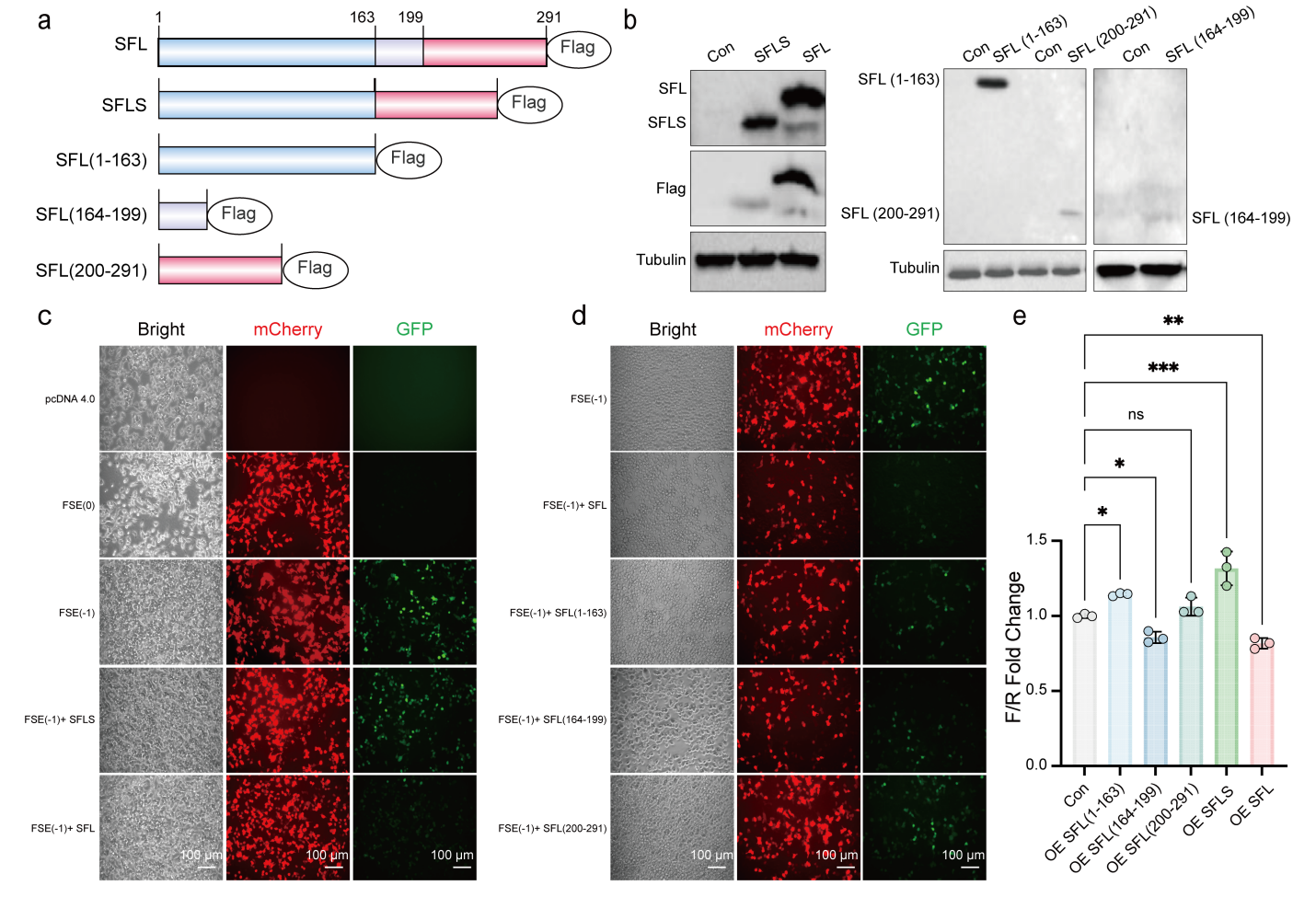


**Supplementary Figure S10. SFL inhibited SARS-CoV-2 frameshifting and interacted with -1 PRF RNA.**

**a** schematic diagram shows Flag-tagged wild-type or truncated mutant SFL. The schematic diagram was generated with [IBS 2.0 (https://ibs.renlab.org/#/server).](http://www.figdraw.com.) **b** HEK293T cells were transfected with Flag-tagged wild-type or truncated mutant SFL vector. The Flag tag was detected using western blotting. The empty vector was used as a negative control. **c–d** Microscopy images of cells cotransfected with the empty vector, Flag-tagged wild-type SFL, SFLS, or truncated mutants of SFL plus the luciferase reporter plasmid in HEK293T cells. The size bar represents 100 μm. **e** HEK293T cells transfected with the pHRF-FSE(-1) luciferase reporter vector were cotransfected with the empty vector, SFLS, SFL wild-type or truncated mutant expression vector. The F/R ratio is used to quantify the level of frameshifting (n=3, ns, not significant, **P < 0.01, and ***P < 0.001). Data are presented as the mean±SEM. Multiple comparisons were performed using ANOVA with Dunnett’s test.

**Supplementary Table S1. Primers used in this study.**

| **Gene Name** | **Primer (5’→3’)** |
| --- | --- |
| *SFL*-F | caagcttggtaccgccaccatgtctcaggaaggtg |
| *SFL*-R | cctcgagttacttatcgtcgtcatccttgtaatcctccctgggcccgcc |
| *SFL*(1-489)-F | caagcttggtaccgccaccatgtctcaggaaggtg |
| *SFL*(1-489)-R | cctcgagttacttatcgtcgtcatccttgtaatcccggaagttgtgccgacacttc |
| *SFL*(490-597)-F | caagcttggtaccgccaccatgggctgggcacagatggggtc |
| *SFL*(490-597)-R | cctcgagttacttatcgtcgtcatccttgtaatcgctgcggcgatccgggtc |
| *SFL*(598-873)-F | caagcttggtaccgccaccatgacccacactcactcctgctc |
| *SFL*(598-873)-R | cctcgagttacttatcgtcgtcatccttgtaatcctccctgggcccgcc |
| *FUBP3*-F | cgggatccgccaccatggcggagctggtgcag |
| *FUBP3*-R | cgctcgagctgctcctggctgtgggcctg |
| *SLBP*-F | cggtaccgccaccatggcctgccgcccgcg |
| *SLBP*-R | cgctcgaggctcatggctgagaagtctctcaag |
| *SLBP-*R46A-F | atgaaatggcgaccagagttaacaaagaaatggca |
| *SLBP-*R46A-R | ctctggtcgccatttcatcttcttcaactgcactt |
| *SLBP-*S94A-F | gacagatgaagctgtcctaatgaggagacagaagca |
| *SLBP-*S94A-R | aggacagcttcatctgtctcaaagtcagccg |
| *RPL10A*-F | cggatccgccaccatggacatcgaggcgctg |
| *RPL10A*-R | cctcgagatataggcgctggggcttgcccatg |
| *RPS3A*-F | cggatccgccaccatggcggttggcaagaac |
| *RPS3A*-R | cctcgagaacagattcttggactggtggttcatatccatcag |
| *RPS14*-F | cgggatccgccaccatggcacctcgaaagggg |
| *RPS14*-R | ccctcgagcagacggcgaccacggcgac |
| SARS-CoV-2-gRNA-F | GACCCCAAAATCAGCGAAAT |
| SARS-CoV-2-gRNA-R | TCTGGTTACTGCCAGTTGAATCTG |
| SARS-CoV-2-gRNA-P | FAM-ACCCCGCATTACGTTTGGTGGAC-BHQ1 |
| SARS-CoV-2-sgRNA-F | CGATCTCTTGTAGATCTGTTCTC |
| SARS-CoV-2-sgRNA-R | ATATTGCAGCAGTACGCACACA |
| SARS-CoV-2-sgRNA-P | FAM-ACACTAGCCATCCTTACTGCGCTTCG-BHQ1 |

**Supplementary Table S2. RNA interference sequences.**

| **siRNAs** | **Sense (5’→3’)** | **Antisense (5’→3’)** |
| --- | --- | --- |
| siNC | UUCUCCGAACGUGUCACGUTT | ACGUGACACGUUCGGAGAATT |
| si*FUBP3* | GGCGAUUUCAACUCUCGAATT | UUCGAGAGUUGAAAUCGCCTT |
| si*SLBP* | GGACCAGAGUUAACAAAGATT | UCUUUGUUAACUCUGGUCCTT |
| si*RPL10A* | CGGGCCUUAUAUAUCAAGATT | UCUUGAUAUAUAAGGCCCGTT |
| si*RPS3A* | CCUGCUAUGUUCAAUAUAATT | UUAUAUUGAACAUAGCAGGTT |
| si*RPS14* | GGAGGAAAUAGGACCAAGATT | UCUUGGUCCUAUUUCCUCCTT |

**Supplementary Table S3. The sequences of RNA and DNA probes.**

| **Name** | **The sequences of RNA probes** |
| --- | --- |
| (-1 FSE)-tRSA | gagacccaagcUUUUUUUAAACGGGUUUGCGGUGUAAGUGCAGCCCGUCUUACACCGUGCGGCACAGGCACUAGUACUGAUGUCGUAUACAGGGCUUUUGACggauccacuaguaacggccgccagugugcuggAAUUGAAAAAAAAAAAAGCCCGGAUAGCUCAGUCGGUAGAGCAGCGGCCUCGACCAGAAUCAUGCAAGUGCGUAAGAUAGUCGCGGGUCGAGGCCGCGUCCAGGGUUCAAGUCCCUGUUCGGGCGCCACUGCAGAAAAAAAAAAAAGaauucugcagau |
| (-1 PRF)-tRSA | gagacccaagcUUCCCAUGCUUCAGUCAGCUGAUGCACAAUCGUUUUUAAACGGGUUUGCGGUGUAAGUGCAGCCCGUCUUACACCGUGCGGCACAGGCACUAGUACUGAUGUCGUAUACAGGGCUUUUGACggauccacuaguaacggccgccagugugcuggAAUUGAAAAAAAAAAAAGCCCGGAUAGCUCAGUCGGUAGAGCAGCGGCCUCGACCAGAAUCAUGCAAGUGCGUAAGAUAGUCGCGGGUCGAGGCCGCGUCCAGGGUUCAAGUCCCUGUUCGGGCGCCACUGCAGAAAAAAAAAAAAGaauucugcagau |
| (-1 PRF Δ2)-tRSA | gagacccaagcUUCCCAUGCUUCAGUCAGCUGAUGCACAAUCGUUUUUAAACGGGUUUGCGGUGUAAGUGCAGCCCGUCUUACACCGUGCGGCACAGGCACUAGUACUGAUGUCGUUUGACggauccacuaguaacggccgccagugugcuggAAUUGAAAAAAAAAAAAGCCCGGAUAGCUCAGUCGGUAGAGCAGCGGCCUCGACCAGAAUCAUGCAAGUGCGUAAGAUAGUCGCGGGUCGAGGCCGCGUCCAGGGUUCAAGUCCCUGUUCGGGCGCCACUGCAGAAAAAAAAAAAAGaauucugcagau |
| (-1 PRF Δ3)-tRSA | gagacccaagcUUCCCAUGCUUCAGUCAGCUGAUGCACAAUCGUUUUUAAACGGGUUUGCGGUGUAAGUGCAGCCCGUCUUACACCGUAUACAGGGCUUUUGACggauccacuaguaacggccgccagugugcuggAAUUGAAAAAAAAAAAAGCCCGGAUAGCUCAGUCGGUAGAGCAGCGGCCUCGACCAGAAUCAUGCAAGUGCGUAAGAUAGUCGCGGGUCGAGGCCGCGUCCAGGGUUCAAGUCCCUGUUCGGGCGCCACUGCAGAAAAAAAAAAAAGaauucugcagau |
| (-1 PRF Δ1＆Δ2)-tRSA | gagacccaagcUUCCCAUGCUUCAGUCAGCUGAUGCACAAUCGUUUUUAAACGGGUUUGCGGCACAGGCACUAGUACUGAUGUCGUUUUGACggauccacuaguaacggccgccagugugcuggAAUUGAAAAAAAAAAAAGCCCGGAUAGCUCAGUCGGUAGAGCAGCGGCCUCGACCAGAAUCAUGCAAGUGCGUAAGAUAGUCGCGGGUCGAGGCCGCGUCCAGGGUUCAAGUCCCUGUUCGGGCGCCACUGCAGAAAAAAAAAAAAGaauucugcagau |
| (-1 PRF Δ2＆Δ3)-tRSA | gagacccaagcUUCCCAUGCUUCAGUCAGCUGAUGCACAAUCGUUUUUAAACGGGUUUGCGGUGUAAGUGCAGCCCGUCUUACACCGUUUUGACggauccacuaguaacggccgccagugugcuggAAUUGAAAAAAAAAAAAGCCCGGAUAGCUCAGUCGGUAGAGCAGCGGCCUCGACCAGAAUCAUGCAAGUGCGUAAGAUAGUCGCGGGUCGAGGCCGCGUCCAGGGUUCAAGUCCCUGUUCGGGCGCCACUGCAGAAAAAAAAAAAAGaauucugcagau |
| (-1 PRF Mutation)-tRSA | gagacccaagcUUCCCAUGCUUCAGUCAGCUGAUGCACAAUCGUUUUUAAACGGGUUUGCGGUGUAAGUGCAGCCCGUCUUACACCGUGCCGCCCAAGCACUAGUACUGAUGUCUUAUACAGGGCUUUUGACggauccacuaguaacggccgccagugugcuggAAUUGAAAAAAAAAAAAGCCCGGAUAGCUCAGUCGGUAGAGCAGCGGCCUCGACCAGAAUCAUGCAAGUGCGUAAGAUAGUCGCGGGUCGAGGCCGCGUCCAGGGUUCAAGUCCCUGUUCGGGCGCCACUGCAGAAAAAAAAAAAAGaauucugcagau |
| -1 PRF RNA truncated mutant I | AUGUCGUAUACAGGGCUUU |
| -1 PRF RNA truncated mutant II | CACAAUCGUUUUUAAACG |
| -1 PRF RNA truncated mutant III | GUGCGGCACAGGCACUAGUACUG |
| ORF1a_positive 1 | GGTGACGCAACTGGATAGAC |
| ORF1a_positive 2 | CTTTCTGTACAATCCCTTTG |
| ORF1a_positive 3 | CCTTGGTTGAATAGTCTTGA |
| ORF1a_positive 4 | CCCCTCTTAGTGTCAATAAA |
| -1 PRF RNA 1 | TGTGCATCAGCTGACTGAAG |
| -1 PRF RNA 2 | CCGCAAACCCGTTTAAAAAC |
| -1 PRF RNA 3 | TGTAAGACGGGCTGCACTTA |
| -1 PRF RNA 4 | AAAGCCCTGTATACGACATC |
| -1 FSE | TTTTTAAACGGGTTTGCGGTGTAAGTGCAGCCCGTCTTACACCGTGCGGCACAGGCACTAGTACTGATGTCGTATACAGGGCTTTTGAC |
| 0 FSE | TTTTTATAAGGGTTTGCGGTGTAAGTGCAGCCCGTCTTACACCGTGCGGCACAGGCACTAGTACTGATGTCGTATACAGGGCTTTTGAC |

Supplementary table S**4: Antibodies and manufacturers**

| Antibody | Company | Catalog No. | Concentrations |
| --- | --- | --- | --- |
| SARS-CoV-2 N | Sinobiological | 40143-MM08 | Western blot: 1:1000,  IF: 1:500 |
| FUBP3 | Abcam | ab181025 | Western blot: 1:1000,  IF: 1:50 |
| SLBP | Abcam | ab181972 | Western blot: 1:1000 |
| SLBP | Abcam | ab221166 | IF: 1:300 |
| RPL10A | Abcam | ab174318 | Western blot: 1:10000,  IF: 1:200 |
| RPS3A | Abcam | ab171742 | Western blot: 1:10000 |
| RPS3A | Proteintech | 14123-1-AP | IF: 1:200 |
| RPS14 | Abcam | ab174661 | Western blot:  1:2000 |
| RPS14 | Proteintech | 67566-1 | IF: 1:800 |
| Flag | CST | #8146 | Western blot:  1:1000 |
| Myc | CST | #2276 | Western blot:  1:1000 |
| HCoV-229E N | Sinobiological | 40640-T62 | Western blot:  1:2000 |
| C19orf66/SFL | Abcam | ab122765 | Western blot:  1:500 |
| ZC3HAV1 | Proteintech | 16820-1-AP | IF: 1:200 |
| Tubulin | Origene | TA503129 | Western blot:  1:2000 |
| goat-anti-mouse HRP IgG | ZSGB‑BIO | ZB-2305 | Western blot:  1:10000 |
| oat-anti-rabbit HRP IgG | ZSGB‑BIO | ZB-2301 | Western blot:  1:10000 |
| Goat Anti-Mouse IgG H&L (Alexa Fluor^®^ 488) | Abcam | ab150113 | IF: 1:500 |
| Goat Anti-Rabbit IgG H&L (FITC) | Abcam | ab6717 | IF: 1:500 |
